# Ethylene glycol metabolism in the oleaginous yeast *Rhodotorula toruloides*

**DOI:** 10.1007/s00253-025-13504-3

**Published:** 2025-05-08

**Authors:** Vittorio Giorgio Senatore, Alīna Reķēna, Valeria Mapelli, Petri-Jaan Lahtvee, Paola Branduardi

**Affiliations:** 1 Department of Biotechnology and Biosciences, University of Milano-Bicocca, Piazza Della Scienza 2, 20126 Milan, Italy; 2 Department of Chemistry and Biotechnology, Tallinn University of Technology, Akadeemia Tee 15, Tallinn, Estonia

**Keywords:** *Rhodotorula toruloides*, Glycolic acid, Ethylene glycol, Xylose, Glucose, Polyethylene terephthalate

## Abstract

**Abstract:**

The agro-food chain produces an impressive amount of waste, which includes not only lignocellulosic biomass, but also plastic, used for both protective films and packaging. Thanks to advances in enzymatic hydrolysis, it is now possible to imagine an upcycling that valorizes each waste through microbial fermentation. With this goal in mind, we first explored the ability of the oleaginous red yeast *Rhodotorula toruloides* to catabolize ethylene glycol (EG), obtained by the hydrolysis of polyethylene terephthalate (PET), in the presence of glucose in batch bioreactor experiments. Secondly, we focused on the physiology of EG catabolism in the presence of xylose as a sole carbon source, and in a mixture of glucose and xylose. Our results show that EG is metabolized to glycolic acid (GA) in all tested conditions. Remarkably, we report for the first time that the consumption of EG improves xylose bioprocess, possibly alleviating a cofactor imbalance by regenerating NAD(P)H. Consumption of EG in the presence of glucose started after the onset of the nitrogen limitation phase, while no significant differences were observed with the control; a 100% mol mol^−1^ yield of GA was obtained, which has never been reported for yeasts. Finally, a putative EG oxidative pathway was proposed by in silico analyses supported with the existing omics data. Our results propose *R. toruloides* as a promising candidate for the production of GA from EG that could be exploited simultaneously for the sustainable production of microbial oils from residual hemicellulosic biomasses.

**Key points:**

*• Ethylene glycol (EG) is not assimilated as a carbon source by Rhodotorula toruloides*

*• With glucose, EG is oxidized to glycolic acid (GA) with a yield of 100% (mol mol* ^*−1*^ *)*

*• With xylose, EG to GA is associated with improved growth and xylose uptake rate*

**Graphical Abstract:**

**Figure:**
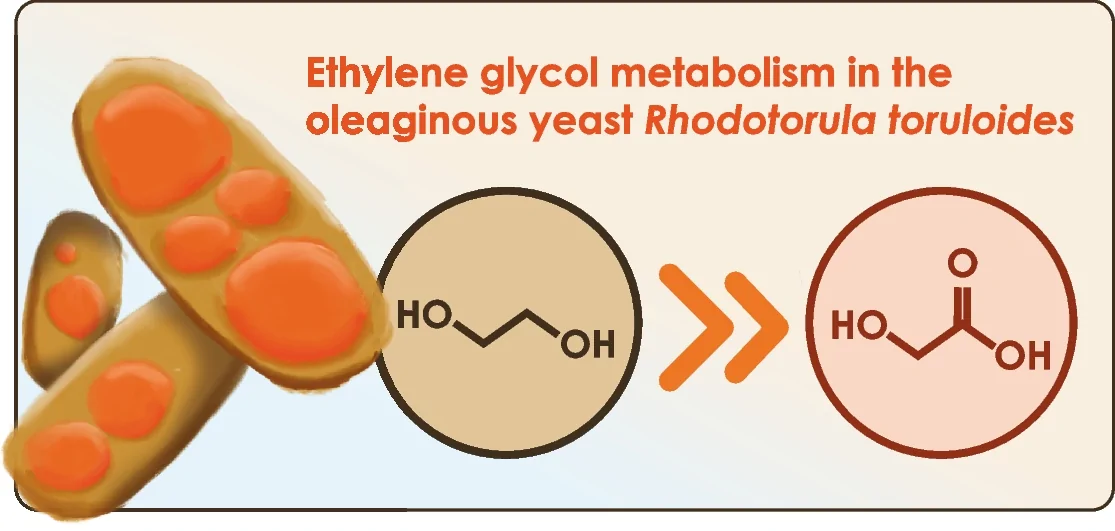

**Supplementary Information:**

The online version contains supplementary material available at 10.1007/s00253-025-13504-3.

## Introduction

Polyethylene terephthalate (PET) is one of the most used thermoplastic polymers on the market, being the third most commonly used plastic in the packaging industry, with a continuous growing demand. PET is vastly used in the food industry, for example in the production of bottles for beverages and agricultural sheets and films (Nisticò 2020). Regarding the agricultural sector in particular, the main application of plastic (polyethylene and PET) is in flexible films, and 17% of the world’s production are multilayer films (Cabrera et al. 2022), which are essential for maintaining the quality and security of many food products. However, recycling rates of plastic packaging are extremely low, especially when considering multilayered films, which combine different materials such as polymers, paper, aluminum and (in)organic coatings (Bauer et al. 2021). At least for PET, sustainable enzymatic hydrolysis solutions are emerging, with the French company CARBIOS (www.carbios.com) being the most famous example.

This aspect of the agricultural production chain is often overlooked or not considered, as many studies just focus on the lignocellulosic waste, which unfortunately is only one side of the agricultural waste. With the final goal to upcycle the most relevant residues in the agrifood sector into high value-added products, we decided to focus our attention on the ability of yeasts — already well characterized and robust microbial cell factories for biorefinery applications (Branduardi 2021) — to catabolize plastic monomers, focusing on ethylene glycol (EG) in particular, one of the two hydrolysis products of PET. Most studies up to now have focused solely on EG catabolism by bacterial species (Zhang et al. 2015; Trifunović et al. 2016; Levin and Balskus 2018; Gao et al. 2022), and studies on yeasts are few and very recent (Carniel et al. 2023; Kosiorowska et al. 2022; Senatore et al. 2024, 2025). In particular, it is reported that different yeast species are able to oxidize EG to glycolic acid (GA), a small α-hydroxy acid used in the cosmetic industry (Salusjärvi et al. 2019) with a market expected to reach $ 565.3 million by 2030 (https://www.grandviewresearch.com/press-release/global-glycolic-acid-market, accessed Aug 20, 2024). The catabolic pathway, however, has only been proposed for yeasts and the genes involved have not been identified yet; there is only a single report of such an attempt (Senatore et al. 2025). Additionally, EG metabolism in yeast has only been studied somewhat in detail for *Yarrowia lipolytica* (Carniel et al. 2023; Kosiorowska et al. 2022) and *Saccharomyces cerevisiae* (Senatore et al. 2024, 2025), in studies focusing on the oxidation of this compound in the presence of glucose, or without sugars as carbon source. These studies, however, lack an in-depth physiology description in controlled conditions.

Red oleaginous yeast *Rhodotorula toruloides* is known for its ability to consume carbon sources released from the hemicellulosic hydrolysates (Bonturi et al. 2017; Lopes et al. 2020b; Monteiro de Oliveira et al. 2021; Brandenburg et al. 2021; Chmielarz et al. 2021) and for its robustness and tolerance in the presence of microbial inhibitory compounds deriving from lignocellulose pre-treatment (Osorio-González et al. 2022b, 2022a). *R. toruloides* is also able to accumulate lipids up to 70% of its biomass from a wide variety of organic substrates (Park et al. 2018; Zhao et al. 2022; Gong et al. 2024), including acids, such as acetic, propionic and butyric acid (Huang et al. 2016; Lopes et al. 2020a; Krikigianni et al. 2022). Moreover, it has been shown that *R. toruloides* is capable of converting EG to GA in a semi-quantitative test in rich medium (Senatore et al. 2024); however, no detailed characterization providing metabolic insights for this catabolism has been shown.

With the long-term goal to develop a cell factory for the upcycling of both lignocellulosic biomass and waste PET hydrolysates, in this study we investigated the *R. toruloides* metabolism of EG in the presence of the most abundant sugars in lignocellulose streams, that is glucose and xylose. Thanks to a thorough physiological characterization in controlled bioreactors, omics data, and bioinformatic analyses, we also propose a putative EG catabolic pathway in *R. toruloides*.

## Materials and methods

### Strain and media composition

*Rhodotorula toruloides* IFO0880 (now called NBRC 0880) was obtained from NBRC culture collection and maintained in 20% (v v^−1^) glycerol at − 80 °C after growth in YPD medium composed of (per liter): yeast extract 10 g (Thermo Fisher Scientific, Rockford, IL, USA), peptone 20 g (Alfa) and glucose 20 g. For YPX, glucose was substituted with (per liter) xylose 20 g. When specified, EG (KeemiaKaubandus, Maardu, Estonia) was added to a final concentration of 150 mM (9.3 g L^−1^), obtaining YPD (YPX) ± EG.

Defined synthetic medium was composed of (per liter): KH_2_PO_4_ 3 g; MgSO_4_·7H_2_O 0.5 g; trace elements 1X (EDTA 30 mg; ZnSO_4_·7H_2_O 9 mg; CoCl_2_·6H_2_O 0.6 mg; MnCl_2_·4H_2_O 2 mg; CuSO_4_·5H_2_O 0.6 mg; CaCl_2_·2H_2_O 9 mg; FeSO_4_·7H_2_O 6 mg; Na_2_MoO_4_·2H_2_O 0.8 mg; H_3_BO_3_ 2 mg; KI 0.2 mg); vitamins 1X (D-biotin 0.10 mg; calcium D-pantothenate 2 mg; nicotinic acid 2 mg; *myo*-inositol 50 mg; thiamine hydrochloride 2 mg; pyridoxal hydrochloride 2 mg; *para-*aminobenzoic acid 0.4 mg) (Verduyn et al. 1992).

For shake flask experiments, glucose and xylose were added to a final concentration of 10 g L^−1^, as described below; to obtain a C/N ratio of 80, (NH_4_)_2_SO_4_ (Fisher bioreagents) was added to a final concentration of 0.275 g L^−1^; for the experiments with both glucose and xylose, (NH_4_)_2_SO_4_ was added to a final concentration of 0.550 g L^−1^; for the experiments with C/N = 8.8, (NH_4_)_2_SO_4_ was added to a final concentration of 5 g L^−1^. When specified, EG was added to a final concentration of 150 mM (9.3 g L^−1^); EG was not considered in the calculations of the C/N ratio. For the experiments in the RTS-8 plus and for the co-utilization of glucose and xylose, the medium was buffered with 100 mM KPO_4_ buffer, pH 6. For bioreactor experiments, glucose was added to a final concentration of 40 g L^−1^; to obtain a C/N ratio of 40, (NH_4_)_2_SO_4_ was added to a final concentration of 2.02 g L^−1^; when specified, EG was added to a final concentration of 75 mM (4.7 g L^−1^); EG was not considered in the calculations of the C/N ratio; to avoid foam formation, antifoam 204 was added to a final concentration of 75 μL L^−1^. For the glucose-limited condition (no nitrogen limitation), glucose concentration was reduced to 20 g L^−1^ and (NH_4_)_2_SO_4_ was added to a final concentration of 5 g L^−1^ (C/N ratio of 8.8).

Unless differently stated, all other reagents were purchased from Sigma-Aldrich Co., St Louis, MO, USA.

### Growth conditions in shake flasks

EG metabolism in the presence of glucose, xylose, glucose and xylose or no additional carbon source was studied in 250-mL shake flasks, in minimal medium ± EG (C/N = 80); EG metabolism in the presence of glucose and xylose was also evaluated with C/N = 8.8.

Seed cultures from YPD plates were grown in sterile glass tubes filled with 2-mL YPD (for the glucose, glucose and xylose, and no carbon source experiments) or YPX (for the xylose experiment) for 8 h; cells were then inoculated for the intermediate inoculum (starting OD_600_ = 0.025 for the cells growing on glucose, and OD_600_ = 0.5 for the cells growing on xylose) in 50-mL glass tubes containing 10 mL of YPD or YPX ± EG, and grown for 16 h. Cells were then harvested, washed with dH_2_O and inoculated in shake flasks (final OD = 0.2) filled with 50 mL of medium under investigation. All growths were performed in a rotary shaker at 200 rpm and 30 °C. Samples were collected at regular time intervals for OD and HPLC analysis. Two independent replicates were performed for each condition. Maximum specific growth rate during the exponential growth phase was estimated by fitting a regression line from the logarithmic transformation of OD values.

### Growth conditions in multi-channel bioreactor

Cultivation in the multi-channel bioreactor RTS-8 plus (SIA Biosan, Riga, Latvia) was performed in minimal medium supplemented with glucose or xylose ± EG (C/N = 80). The pH, pO2 and optical density were measured throughout time. For the cultivations with glucose, a second set of experiments was performed by adding 100 mM KPO_4_ buffer, pH 6 to limit the pH drop.

Seed cultures from YPD plates were grown in sterile glass tubes filled with 2 mL YPD or YPX for 8 h; cells were then inoculated for the intermediate inoculum (starting OD = 0.025 for the cells growing on glucose, and OD = 0.5 for the cells growing on xylose) in 50-mL glass tubes containing 10 mL of YPD or YPX, and grown for 16 h. Cells were then harvested, washed with dH_2_O and inoculated in TubeSpin® Bioreactor tubes with pH and dO_2_ sensors (final OD = 0.2) filled with 10 mL of medium under investigation; growth was performed in the RTS-8 plus multi-channel bioreactor.

Samples were collected at regular time intervals for OD, pH and HPLC analysis; live %DO (percentage of dissolved oxygen) was used to estimate the maximum specific growth rate, as well as for the determination of the exponential growth and nitrogen limitation phase intervals.

### Bioreactor cultivations

#### Growth conditions in bioreactor

*R. toruloides* was grown in 1 L stirred tank bioreactors (Applikon Biotechnology, Delft, The Netherlands) in a batch cultivation regime. pH was controlled by the addition of 2 mol L^−1^ KOH. Dissolved oxygen was maintained not lower than 25% at 1 vvm airflow by regulating the stirring speed. CO_2_ and O_2_ outflow gas composition was measured using off-gas sensors (BlueInOne, BlueSens, Herten, Germany). Data collection and processing was performed with Lucullus PIMS Lite v3.7.4 software (Getinge, Göteborg, Sweden).

Seed cultures from YPD plates were grown for 12 h in sterile glass tubes in 10 mL YPD; cells were then inoculated for the intermediate inoculum (starting OD = 0.5) in 250-mL baffled shake flasks containing 50 mL of YPD and grown for 20 h. The pre-inocula were performed in a rotary shaker at 200 rpm and 30 °C. For the inoculum, cells were harvested, washed with sterile dH_2_O, and used to inoculate the bioreactor (starting OD = 0.5). Cells were grown in Delft medium ± EG, as described above.

Samples were collected at regular time intervals for OD_600_, cell dry weight (CDW) measurement, total protein content, lipidomics, nitrogen quantification, and HPLC analysis. Three independent experiments were performed for each condition. Maximum specific growth rate during the nitrogen limitation phase was estimated by fitting a regression line from the logarithmic transformation of both OD and CDW values.

#### Quantification of lipids

Cell broth containing at least 12 mg of dry cell weight was withdrawn from bioreactor to a 15 mL tube, pelleted (5000 g, 10 min, 4 °C) and stored at − 80 ℃. Prior to analysis, the pellet was lyophilized and stored at − 20 ℃. Fatty acids of freeze-dried biomass samples were extracted and derivatized by using one-step extraction and derivatization method as described by Sukhija and Palmquist (Sukhija and Palmquist 1988), with internal standard heptadecanoic acid (17:0) in toluene (5 mg mL^−1^). Fatty acids were analyzed using Agilent (Santa Clara, CA, USA) 6890 A gas chromatograph with flame ionization detector. Analysis was done with the quartz capillary column with liquid phase CP-Sil 88 (100 m × 0.25 mm), temperature programmed from 70 to 180 ℃ at 13 ℃ min^−1^, held for 40 min, then 180 to 225 ℃ at 5 ℃ min^−1^, held for 15 min, using hydrogen as the carrier gas (flow rate 30 mL min^−1^), air flow rate 300 mL min^−1^. Fatty acids were identified by comparison of their retention times with the retention time of commercial standards Supelco 37 Component FAME Mix (Sigma-Aldrich Co., St. Louis, MO, USA), Nu-Chek Prep CLC 603 and Nu-Chek Prep CLC 428 (Nu-Chek Prep Inc., Elysian, MN, USA). The results are presented as the content of individual fatty acids in g/100 g of total fatty acids (the same as %) or the amount of each fatty acid in 100 g of sample. Total fatty acids (%) were calculated as the sum of individual fatty acids. To provide the TAG equivalent quantity, the sum was divided by 0.9.

#### Quantification of intracellular proteins

Cell broth containing 600 μg of dry cell weight was withdrawn from bioreactor to a 2 mL tube and pelleted (21,000 g, 5 min). Pellet was washed once with dH_2_O and stored at − 20 °C until further analysis. Protein extraction was performed with the commercially available Y-PER (Yeast Protein Extraction Reagent) (Thermo Fisher Scientific, Rockford, IL, USA), according to the manufacturer’s instructions; to avoid protein degradation, a protease inhibitor cocktail (Halt Protease Inhibitor Cocktail EDTA-Free, Thermo Fisher Scientific, Rockford, IL, USA) was added to the Y-PER reagent. Four rounds of repeated extractions were performed, until no proteins were detected in supernatant. Total protein content was quantified using a commercially available colorimetric assay kit (Micro BCA Protein Assay Kit, Thermo Fisher Scientific, Rockford, IL, USA). Protein concentration was determined using a calibration curve of bovine serum albumin standard of linear range dilutions from 0.5 to 200 μg mL^−1^. Assays were performed in duplicate for each sample. Only samples corresponding to the end of the nitrogen limitation (Nlim) phase were analyzed.

#### Nitrogen quantification

Cell broth was withdrawn from bioreactor to a 1.5 mL tube and centrifuged (21,000 g, 5 min). Supernatant was filtered (0.22 μm) and stored at − 20 ℃ until further analysis. Upon analysis, samples were thawed and diluted when necessary. Concentration of NH_4_^+^ was determined with the commercially available Urea/Ammonia Assay Kit (Rapid) (Megazyme Ltd, Wicklow, Ireland) by reading the absorbance at 340 nm with a multiplate reader (FilterMax F5, Molecular Devices, San Jose, CA, USA) and using a calibration curve of (NH_4_)_2_SO_4_ in linear range dilutions from 0.0 mg L^−1^ to 275 mg L^−1^ (corresponding to concentrations of NH_4_^+^ of 0.0 mg L^−1^ to 75 mg L^−1^). Samples chosen for analysis correspond to the beginning of the fermentation, to the switch to the Nlim phase and to the end of the Nlim phase.

### Quantification of dry cell mass and extracellular metabolites

Extracellular metabolites glucose, xylose, ethylene glycol, glycolic acid, acetate and glycerol were quantified using HPLC (LC-2050 C Plus, Shimadzu, Kyoto, Japan) equipped with a refractive index detector and a variable wavelength detector. Prior to analysis, cells were removed by centrifugation (21,000 g, 5 min), and the supernatant filtered (0.22 μm) and diluted when necessary. The HPLC was equipped with the Aminex HPX-87H 300 × 7.8 mm (Bio-Rad, Hercules, CA, USA); 10 μL of sample were injected in the column. The mobile phase was H_2_SO_4_ 5 mM, at a flow of 0.6 mL min^−1^; column temperature was set to 60 °C. Separated components were detected by the refractive index detector, and by the variable wavelength detector set at 210 nm. Peaks were identified by comparison with reference standards dissolved in ultrapure H_2_O (18 MΩ) (Milli-Q Ultrapure Water System, Merck, Darmstadt, Germany). Calibration curves for peak quantification were prepared in a range between 0.625 and 40.0 g L^−1^.

Dry cell mass was calculated from optical density measurements using calibration coefficient 0.3. Calibration was performed gravimetrically on the dry cell mass four times during the cultivation.

### Statistical analysis

The experiments were performed with three independent replicates. GraphPad PRISM 10.1.0 was used for the statistical analysis of cultivation parameters, performed using a two-tailed, unpaired, heteroscedastic Student’s *t*-test.

### Genome-scale metabolic modeling

Flux Balance Analysis (FBA) was used to predict in silico intracellular metabolic flux data for *R. toruloides* NBRC 0880 using the genome-scale metabolic network Rt_IFO0880 (Kim et al. 2021) with the Cobrapy toolbox (Ebrahim et al. 2013) using Gurobi solver version 11.0 (Gurobi Optimization Inc., Houston, Texas, USA). The model was improved by adding ethylene glycol (C_2_H_6_O_2_) as a metabolite to the extracellular space and cytosol, adding ethylene glycol cytosolic transport, and ethylene glycol oxidase—dehydrogenase to the cytosol. To allow the model to predict the production of either NADPH, or NADH during the ethylene glycol consumption, two oxidation reactions were added, homologous to *S. cerevisiae YLL056C* and *GRE2*. Model calculations were performed with parsimonious FBA (pFBA) (Lewis et al. 2010) optimizing for the specific growth rate and followed by flux variability analysis with random sampling of the solution space with 2000 sampling iterations at 10% variability from predicted specific growth rate. Specific substrate uptake rates obtained from yeast cultivation in this study were used to constrain the consumption of xylose, glucose or ethylene glycol by setting fixed flux constraints where the lower and upper bound were set equal to the measured flux value (Supplementary Table S1). Measured specific glycolic acid production rates from this study were used to constrain its production rate by setting a fixed flux constraint for both bounds equal to the measured flux value (Supplementary Table S1). For the analysis, fluxes were normalized by dividing absolute flux with the specific substrate uptake rate (i.e., xylose or glucose). For calculations, median flux, mmol/gCDW/h, obtained from 2000 iterations of random sampling of the solution space, was used. Model curation, pFBA, random sampling script and simulation results were uploaded on a Github repository EG-bio-upcycling: https://github.com/bioengtaltech/EG-bio-upcycling.

### Bioinformatics analysis

To identify potential enzymes of the EG catabolic pathway in *R. toruloides*, protein sequences of gene candidates identified from the literature were retrieved from NCBI and used as a query in a JGI Mycocosm Blast search of the *R. toruloides* IFO0880 reference genome v4.0 (Coradetti et al. 2018) protein database (Rhoto_IFO0880_4_GeneCatalog_proteins_20170509.aa) with default parameters: “E = 1e5; Filter = True BLOSUM62” and NCBI protein database Non-redundant protein sequences (nr) with search limited to records that include: *Rhodotorula toruloides* NP11 (taxid:1130832), *Rhodotorula toruloides* (taxid:5286); default algorithm parameters were utilized.

Clustal Omega Multiple Sequence Alignment (MSA) tool was utilized for sequence alignments (Madeira et al. 2024); default algorithm parameters were utilized.

## Results

### Ethylene glycol catabolism in the presence of glucose

To investigate EG metabolism in *R. toruloides*, we decided to focus our attention on the cultivations in the presence of glucose, as many yeasts have a distinct behavior in the presence of this sugar. Moreover, glucose is one of the most abundant hexose sugars in lignocellulosic hydrolysates (Monteiro de Oliveira et al. 2021). We started our investigations by assaying the ability of *R. toruloides* to metabolize EG in shake flask experiments with standard glucose concentration (10 g L^−1^), and we compared growth curves with a control condition in which EG was not supplemented. As *R. toruloides* is known as oleaginous yeast and microbial oils could be one of the target products, lipid synthesis was induced by setting a C/N ratio of 80 in the medium (Pinheiro et al. 2020) (see “Materials and methods” section). The carbon from EG was not considered in the calculations of the C/N ratio.

**Fig. 1:**
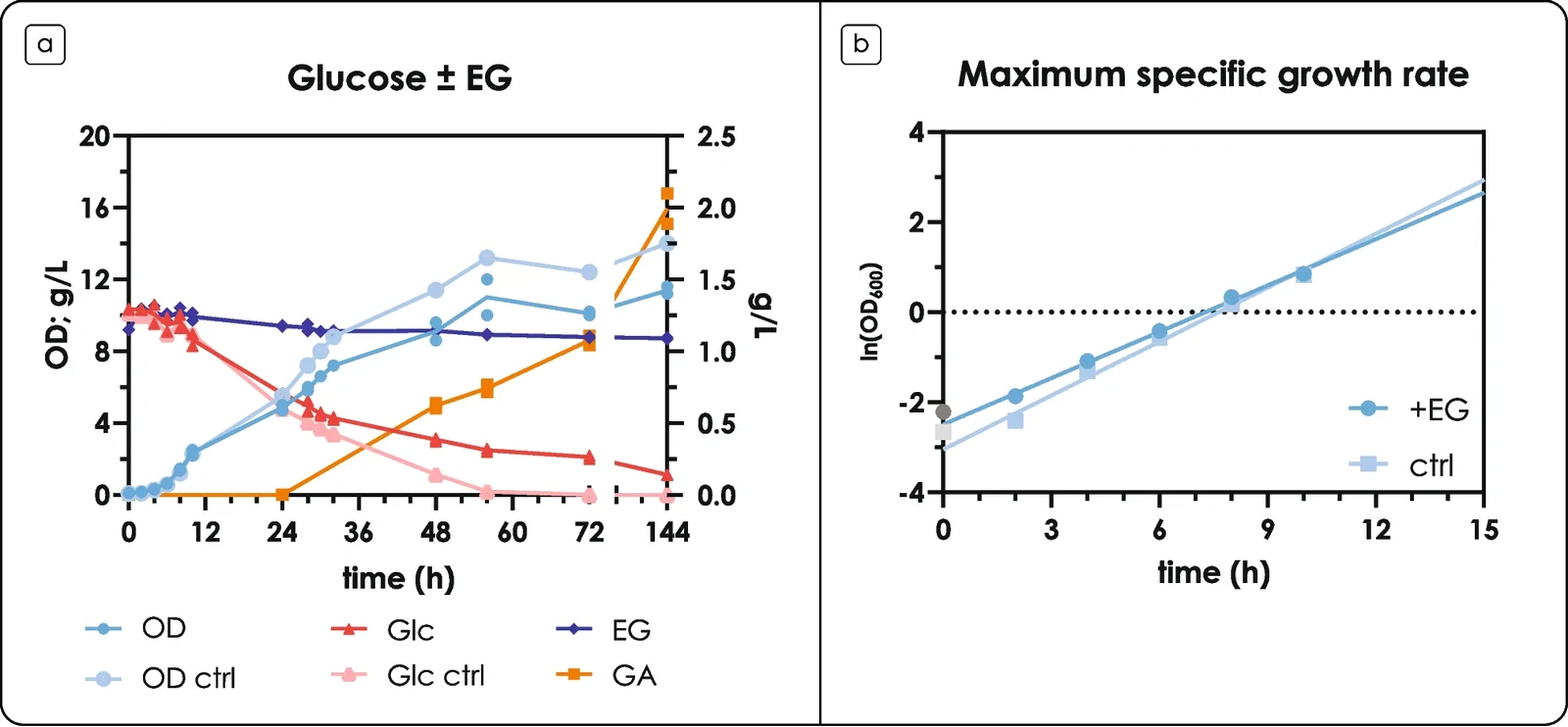
Fermentation profiles on glucose, in the presence of ethylene glycol (EG) in shake flasks. (a) Fermentation profiles in the presence of glucose ± EG. The left y-axis shows OD (blue, circles), and glucose (red, triangles) and EG (dark blue, diamonds) concentration in g L^−1^; the right y-axis shows GA (orange, squares) concentration in g L^−1^. (b) Growth rates in the presence of glucose ± EG. The y-axis shows the natural logarithm of the OD and the calculated regression line; the slope of the line was used to represent the growth rate. Data corresponding to the control condition (ctrl) have a lighter shade of the color. Glc: glucose. Values are the mean of two independent experiments

By looking at substrate consumption and biomass generation over time (Fig. 1a), two distinct phases can be observed. First, during the exponential phase (until 10 h from the inoculum, at least, Fig. 1b), no differences in the specific growth rate could be observed compared to the control condition (no EG) and EG was not consumed (0.34 ± 0.02 h^−1^ vs 0.40 ± 0.08 h^−1^). After 24 h (and presumably the onset of the nitrogen limitation (Nlim) phase), EG started to be consumed, and GA was produced, reaching a final concentration of 1.99 ± 0.11 g L^−1^ and a molar yield of 98.83% mol mol^−1^. Overall, the presence of EG apparently affected *R. toruloides* growth, as final OD was lower, and glucose consumption during the second phase of cultivation (Nlim) was slower compared to control; in particular, glucose was not depleted even after 144 h of cultivation, while glucose depletion already occurred around 56 h in the control. This behavior could be explained by the drop in pH (data not shown) caused by the production of GA, as no pH buffer system was used in the experiment. To confirm the hypotheses, the cultivation was repeated in a multi-channel falcon tube bioreactor RTS-8 plus with real-time OD and %DO monitoring; buffered media (pH 6) were included, too. The results are shown in Supplementary Fig. S1 (panels A, B and C). The effect of pH is reflected in lower biomass accumulation: the presence of EG (i.e., the production of GA) yielded a lower final OD both with and without buffer (Supplementary Fig. S1**a**); additionally, no significant differences in the maximum specific growth rates were observed, in accordance with the first results. In the absence of buffer, the pH in the presence of EG dropped to 2 at the end of the fermentation (Glc.EG), as opposed to pH 3 measured for the control condition (Glc.ctrl) (Supplementary Fig. S1**b**). In the presence of a buffer system, the pH remained unchanged for the control (Glc.B.ctrl), while it only lowered to pH 5 in the presence of EG (Glc.B.EG). The %DO data also allowed to accurately distinguish the exponential phase from the Nlim phase, as well as the depletion of the carbon source (Supplementary Fig. S1**c**). It is worth noting that buffered media allowed faster glucose consumption, and that GA was produced even after glucose depletion in Glc.B.EG (Supplementary Fig. S1**b**).

**Fig. 2:**
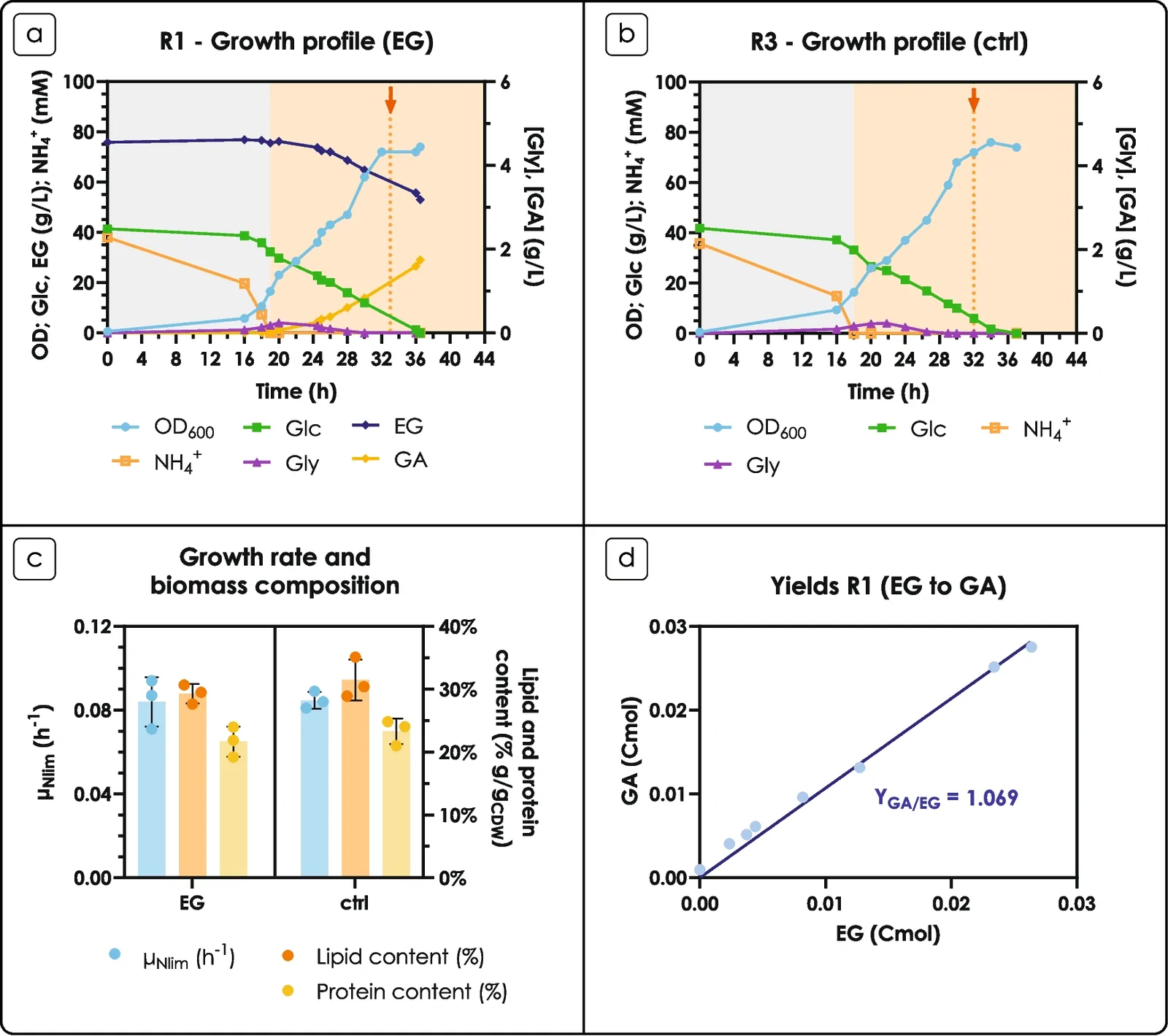
Quantitative physiology of EG metabolism in the presence of glucose. (a) Fermentation profile of *R. toruloides* in the presence of glucose and EG. (b) Fermentation profile of *R. toruloides* in the control condition (no EG). For panels (a) and (b), the left y-axis shows OD (blue, circles), glucose (green, squares) and EG (dark blue, diamonds) concentration in g L^−1^, and ammonium concentration in mM; the right y-axis shows glycerol (purple, diamonds) and GA (yellow, diamonds) concentration in g L^−1^. The exponential phase is shaded in gray, while the Nlim phase is shaded with orange; the arrow indicates the time of sampling for total protein and lipid profile analyses. (c) Comparison of specific growth rate and biomass composition in the presence and absence of EG during the Nlim phase. The left y-axis shows the calculated growth rates (μ_Nlim_) in h^−1^ (blue); the right y-axis shows lipid (orange) and protein (yellow) content (% g g_CDW_^−1^). (d) Yield of glycolic acid on consumed ethylene glycol (Cmol Cmol^−1^); the slope of the line is the calculated conversion yield of EG to GA. For panels (a), (b), and (c) only one replicate is shown for clarity, as *R. toruloides* IFO0880 showed slightly different lag phase durations among replicates; Supplementary Fig. S3, S4, and S5 show the full set of data. For panel (d) values are the mean ± standard deviation of three independent experiments

To obtain more detailed physiology data, *R. toruloides* was grown in minimal synthetic medium in controlled bioreactors. First, *R. toruloides* was grown without nitrogen limitation (Supplementary Fig. S2) to assess if EG consumption and GA production are linked to a starvation condition and therefore do not happen under balanced conditions during the exponential growth phase, when no limitation occurs. Upon a number of tests (data not shown), a medium with 40 g L^−1^ glucose and a C/N ratio of 40 was selected as the best condition to measure GA production and EG consumption. Compared to the shake flask experiments, the lower C/N ratio allowed to have a good balance between the duration of the exponential and the Nlim phases, while the increase to 40 g L^−1^ of glucose allowed to have enough biomass at the onset of the Nlim phase for obtaining quantitative data on lipid production. The results are shown in Fig. 2. The cultivation profiles for all replicates are shown in Supplementary Fig. S3 (EG) and Supplementary Fig. S4 (control), along with CO_2_ production and growth rates; yields of GA from EG for each replicate are shown in Supplementary Fig. S5, along with the biomass (X) and CO_2_ yields on substrate (S) for carbon balance calculation (Table 1).

**Table 1:** Carbon balance of the Nlim phase in the absence and presence of EG. The table shows the yields in Cmol Cmol^−1^ (X/S and CO_2_/S) for the calculation of the carbon balance; yields of EG/S and GA/S were not considered as their net sum is 0. Indeed, 100% of EG is converted to GA (see main text for more details). It is worth noting that it was not possible to close the carbon balance, for both conditions. This might be due to a small production of TCA cycle-derived organic acids, which could be observed (but not quantified) from the chromatograms at 210 nm; a similar pattern was observed for both conditions (data not shown). Values are the mean and standard deviation of three independent experiments

		Carbon balance	Y_X/S_ [Cmol/Cmol]	Y_CO2/S_ [Cmol/Cmol]	Y_GA/EG_ [Cmol/Cmol]
**Ctrl**	**avg**	92.8%	0.562	0.366	-
	**sd**	2.2%	0.009	0.020	-
**EG**	**avg**	93.1%	0.554	0.377	1.010
	**sd**	1.1%	0.009	0.006	0.042

No significant differences in the growth phenotype — apart from the obvious production of GA on the onset of Nlim phase — were observed with the control condition in which EG had not been added. The cultivation profiles obtained in the two conditions (Fig. 2a and Fig. 2b) were very similar: during the Nlim phase, no differences were observed in specific growth rate (0.084 ± 0.010 h^−1^ and 0.084 ± 0.003 h^−1^), biomass and CO_2_ yields, and respiratory quotient (RQ) (Fig. 2c; Table 2). This evidence supports our hypothesis that the differences observed in shake flasks were due to non-controlled pH of the culture. As expected, the measured specific growth rate during the Nlim phase was more than two times lower as compared to the exponential phase (see Fig. 1b). Next, to evaluate the potential effect on lipid production, we analyzed total lipid content and fatty acid profile from samples taken at the end of cultivation. As lipid synthesis is associated with reallocation of resources from protein synthesis machinery, we also evaluated the total protein content in the cell mass sampled at the end of cultivation. The same protein content and the same lipid content and profile resulted from the two conditions (Fig. 2c; Supplementary Table S3); all comparisons had a *p*-value > 0.05. These results, while unexpected, suggest that EG metabolism in the presence of glucose does not affect *R. toruloides*’s metabolism in a significant way. Proteomics data are necessary to strongly support this conclusion, but such analysis falls out of the scope of the current study.

**Table 2:** Calculated yields, respiratory quotient (RQ), and protein and lipid content. Values are the mean and standard deviation of three independent experiments

		Y_X/S_ [g_CDW_/g]	Y_CO2/X_ [mmol/g_CDW_]	Y_O2/X_ [mmol/g_CDW_]	RQ	Protein content [g/g_CDW_]	Lipid content [g/g_CDW_]	Y_GA/EG_ [mol/mol]	Y_GA/EG_ [g/g]
**Ctrl**	**avg**	0.42	29.88	23.32	1.29	0.23	0.31	-	-
	**sd**	0.01	1.02	1.12	0.06	0.02	0.03	-	-
**EG**	**avg**	0.41	30.63	22.57	1.36	0.22	0.29	1.01	1.23
	**sd**	0.02	1.96	1.44	0.01	0.02	0.01	0.03	0.04

In terms of EG consumption and GA production, 1.68 ± 0.06 g L^−1^ of GA were produced during the Nlim phase, with a volumetric productivity of 0.13 ± 0.01 g L^−1^ h^−1^. While titer and productivity are lower than what is reported with other yeasts (Carniel et al. 2023; Senatore et al. 2024), the obtained yield of GA on consumed EG during glucose co-consumption was 100% (1.01 ± 0.03 mol mol^−1^) (Fig. 2d; Table 2). This result is interesting, as GA/EG yields in other yeasts are typically lower. In particular, Carniel and colleagues obtained a maximum yield of GA on consumed EG of 74% mol mol^−1^ with *Y. lipolytica* (Carniel et al. 2023); similarly, Senatore and colleagues reported a yield of 77% mol mol^−1^ with *Scheffersomyces stipitis* (Senatore et al. 2024). The highest yield was obtained with *S. cerevisiae* (94% mol mol^−1^) after two rounds of optimization with design of experiment (Senatore et al. 2024). These observations suggest that, at least in the presence of glucose, *R. toruloides* is not able to further oxidize GA to GOX, making it an excellent choice when considering a biorefinery for the conversion of EG to GA.

### Ethylene glycol catabolism in the presence of xylose or no additional carbon source

We also decided to assay the ability of *R. toruloides* to metabolize EG in the presence of xylose, the second most abundant sugar in hemicellulosic hydrolysates, or without sugar addition to the medium, in order to evaluate the ability of *R. toruloides* to use EG as the only carbon source for growth or energy metabolism. Similarly to the experiment with glucose, a C/N ratio of 80 was selected to induce lipid accumulation.

**Fig. 3:**
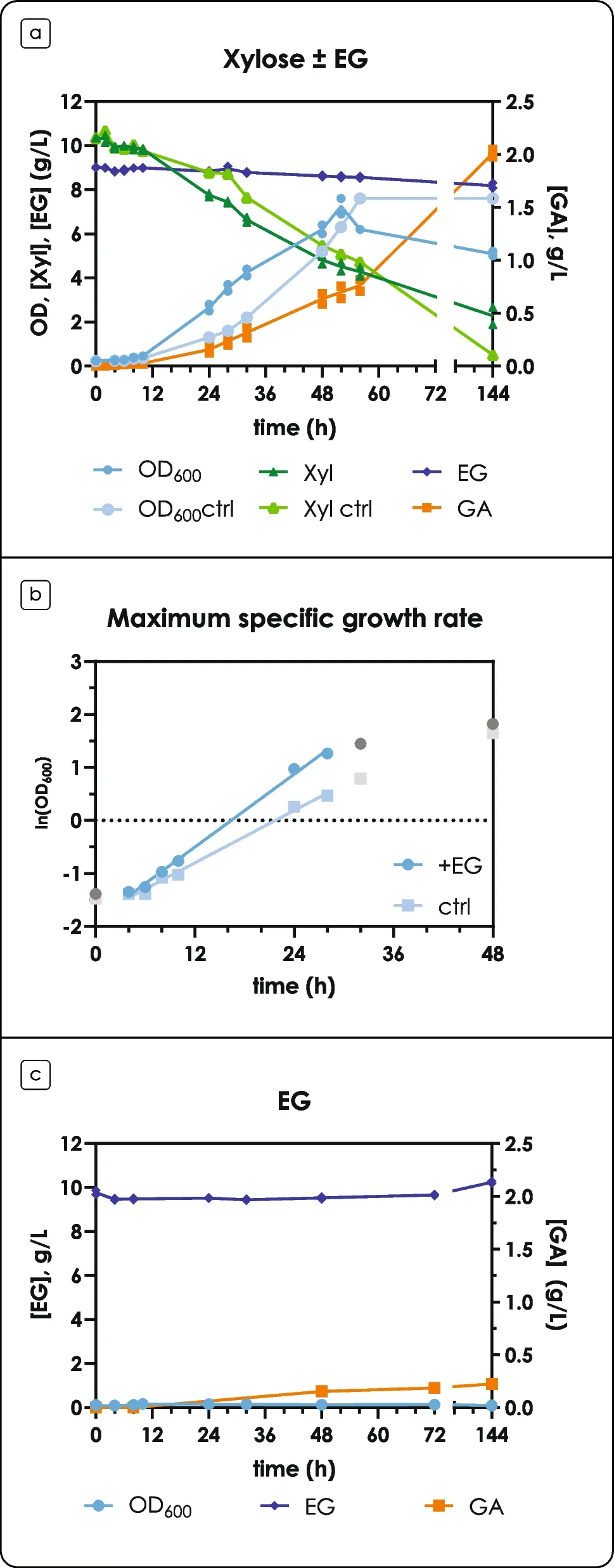
Fermentation profiles on xylose or no carbon source in the presence of ethylene glycol (EG) in shake flasks. (a) Fermentation profiles in the presence of xylose ± EG. The left y-axis shows OD (blue, circles), and xylose (green, triangles) and EG (dark blue, diamonds) concentration in g L^−1^; the right y-axis shows GA (orange, squares) concentration in g L^−1^. Data corresponding to the control condition (ctrl) have a lighter shade of the color. Xyl: xylose. Values are the mean of two independent experiments. (b) Growth rates in the presence of xylose ± EG. The y-axis shows the natural logarithm of the OD and the calculated regression line; the slope of the line was used to represent the growth rate. Data corresponding to the control condition (ctrl) have a lighter shade of the color; points that were excluded from the fitting are shaded in gray. (c) Fermentation profile in the presence of EG as the sole carbon source. The left y-axis shows OD (blue, circles), and EG (dark blue, diamonds) concentration in g L^−1^; the right y-axis shows GA (orange, squares) concentration in g L^−1^. Values are the mean of two independent experiments

In the presence of xylose, GA was detected after 2 h from the inoculum (Fig. 3a) and a co-consumption of xylose and EG occurred throughout the cultivation (including at late stages of fermentation, presumably the Nlim phase). The final concentration of GA at 144 h was 2.01 ± 0.03 g L^−1^. Interestingly, during the exponential growth phase, the maximum specific growth rate was significantly higher in the presence of EG (0.11 ± 0.01 h^−1^ vs 0.08 ± 0.01 h^−1^, *p* < 0.0001, Fig. 3b), with almost double the amount of xylose consumed in the presence of EG towards the end of the exponential phase (2.90 g L^−1^ vs 1.63 g L^−1^, at 28 h). The maximum specific growth rate was more than three times lower as compared to the glucose condition. Similar observations have been reported in previous studies (Reķēna et al. 2023). Tracking of the pH by growing the cells in a multi-channel bioreactor RTS-8 plus with real-time OD and %DO monitoring confirmed the increase in maximum specific growth rate in the presence of EG (0.22 ± 0.01 h^−1^ vs 0.08 ± 0.01 h^−1^, *p* < 0.001, Supplementary Fig. 1a); moreover, a longer lag phase was observed in the control without EG. In the presence of EG, the pH dropped from 5 to 3 already after 24 h, and reached pH 2 at the end of the cultivation; in the control condition, the pH drop was more gradual, and reached a final value of 3 (Supplementary Fig. 1b), however not having an effect on final biomass OD and xylose consumption. The %DO monitoring (Supplementary Fig. 1c) showed that the nitrogen starvation in presence of EG commenced after 12 h, therefore we used the experimental data to calculate yields (on consumed EG) and specific production and consumption rates during each growth phase. The results suggested 74.3 ± 9.4% GA/EG mol mol^−1^ during the exponential growth phase, 159.8 ± 20.4% GA/EG mol mol^−1^ during the Nlim phase (Supplementary Table S1), suggesting that not only *R. toruloides* cannot catabolize EG into biomass, but also that GA might be produced from other reactions. Notably, GA was never observed in any of the control conditions, i.e., when EG had not been added to the medium.

**Fig. 4:**
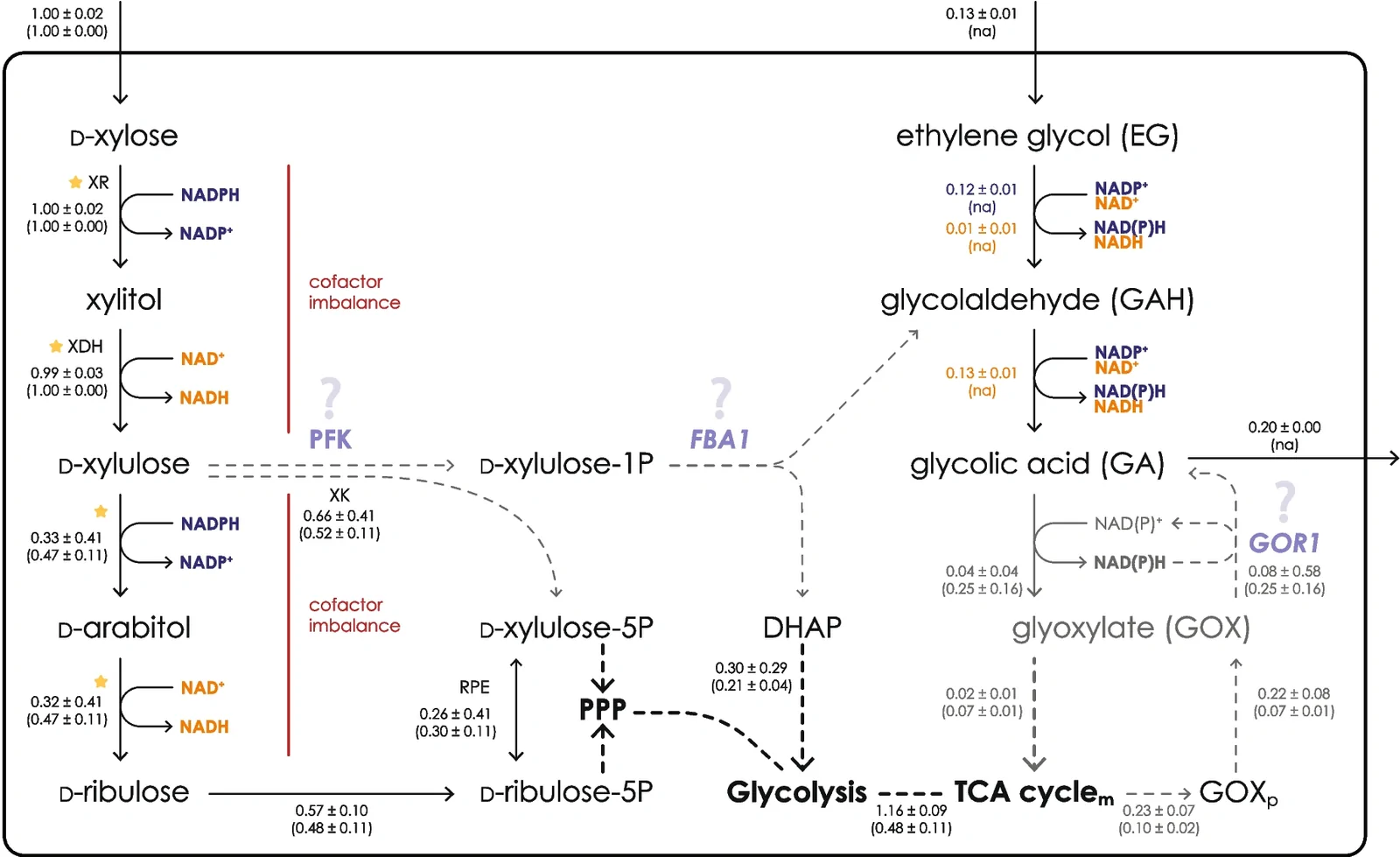
Xylose and (proposed) ethylene glycol (EG) metabolism in *Rhodotorula toruloides*. Xylose catabolism in *R. toruloides* requires two oxidation and two reduction reactions, which could either be NAD- or NADP-dependent, generating a cofactor imbalance limiting xylose utilization. EG oxidation to glycolic acid (GA) regenerates two equivalents of NAD(P)H for every molecule of EG, thus possibly relieving the cofactor imbalance of xylose catabolism. Two additional pathways are proposed. (1) The intermediate D-xylulose might be phosphorylated to D-xylulose-1P, later cleaved into dihydroxyacetone phosphate (DHAP) and glycolaldehyde (GAH); (2) glyoxylate (GOX) produced from the TCA cycle can be reduced to glycolic acid (GA) in the cytosol and in the mitochondria by *GOR1*. These hypotheses explain why the obtained yield of GA is greater than 100% mol mol^−1^ when in presence of xylose. The numbers on the reaction indicate the median of normalized predicted flux of Xyl.EG condition and in parentheses control Xyl condition; when necessary, the compartment of the metabolite is indicated with a subscript (m: mitochondrion; p: peroxisome). All fluxes are available in Supplementary Table S2. XR: xylose reductase; XDH: xylitol dehydrogenase; PFK: phosphofructokinase; *FBA1*: fructose-bisphosphate aldolase; *GOR1*: glyoxylate reductase; DHAP: dihydroxyacetone phosphate; PPP: pentose phosphate pathway; RPE: ribulose 5-phosphate 3-epimerase; XK: xylulokinase. Flux values represent model Rt_IFO0880 reactions (alphabetically): AGTim; DABT2D; DABT4D; EX_eg_e; EX_glyclt_e; EX_xyl__D_e; GAPD; GLYCDO1p; GLYCLTt; GLYCLTDy; GLYCLTDxm; GLYCLTt; GLYCLTtm; GLXtp; ICLp, RPE, T_eg; TPI; XYLTD_D; XYLK; XYLR; XYLt. Standard deviation is calculated from random sampling results (*n* = 2000). Colors of the fluxes represent which cofactor was provided to the simulation (orange: NAD, blue: NADP). Stars along the reaction indicate that multiple genes encode the same enzymatic activity in *R. toruloides*

To better describe this aspect, genome-scale modeling with the Flux Balance Analysis was used to predict in silico intracellular metabolic fluxes during xylose consumption in the presence of EG. To understand how GA might be produced from other reactions, we compared it with a simulation on glucose in presence of EG equimolar to GA. For a comparison, control simulations with no EG presence on xylose or glucose were performed. Genome-scale models comprise stoichiometrically reconstructed enzymatic and transport reactions from gene-protein relationship (GPR) information, allowing to make quantitative predictions of intracellular flux rates using physiology data obtained in this study (Supplementary Table S1). Fixed exchange flux constraints applied to xylose, glucose, EG and GA exchange helped to ensure realistic model predictions. Regarding EG assimilation pathway, the model predicted the flux via NADP-dependent EG dehydrogenase on either xylose or glucose as the substrate (92 ± 1% or 100% of EG, respectively) over the NAD-dependent EG dehydrogenase. Furthermore, the model predicted 23 ± 7% of carbon from xylose (normalized flux 0.23) channeled from the TCA cycle via glyoxylate shunt to the peroxisome (isocitrate lyase, *ICL*p, converting isocitrate to glyoxylate), followed by glyoxylate transport to the cytosol and a subsequent conversion of glyoxylate into GA by glyoxylate reductase (GLYCLTD, *GOR1*) (Fig. 4). The *GOR1* flux prediction was 8% of carbon from xylose with a flux variability of 58%. Moreover, the excess flux from the glyoxylate shunt was not channeled back to the TCA cycle via alanine-glyoxylate aminotransferase (AGTim), as in the case of control condition (Xyl) simulation, but instead secreted as glyoxylate (6 ± 6% of carbon from xylose, not shown on Fig. 4). However, no unidentified peaks on HPLC chromatograms were found. Peroxisomal glyoxylate cycle activity was predicted also in the glucose condition, moreover it occurred irrespective of the EG presence (44 ± 21% and 67 ± 80%, respectively) and taking into account that the EG and GA were constrained at equimolar specific rates. All results are available in Supplementary Table S2.

Xylose utilization by yeast generally requires a NADPH-dependent reduction of xylose to xylitol, and a subsequent NAD^+^-dependent oxidation to xylulose, generating NADH instead of NADPH, leading to a cofactor imbalance (Monteiro de Oliveira et al. 2021) (Fig. 4); a similar scenario is possible in the two subsequent reactions (reduction to D-arabitol, oxidation to D-ribulose) (Adamczyk et al. 2023). While a pathway for EG oxidation to GA in yeast has only been proposed (Senatore et al. 2024, 2025), two NAD(P)H molecules are generated for every molecule of GA produced, which might partially relieve the cofactor imbalance (and/or push the xylose catabolism) by regenerating some NADPH and explaining the improved fitness of the strain in the presence of EG. However, the reverse reaction catalyzed by Gor1 consumes one NADPH molecule. Alternatively, the additional GA might be produced from an intermediate of the XR-XDH pathway, due to one or more enzymatic activities which are expressed only in the Nlim phase. In a study with *S. cerevisiae*, Uranukul and colleagues demonstrated that *S. cerevisiae*’s phosphofructokinase (PFK) is able to natively catalyze the phosphorylation of D-xylulose to D-xylulose-1P (Uranukul et al. 2019). D-xylulose-1P can then undergo cleavage between the C3 and C4 position producing dihydroxyacetone phosphate (DHAP) and glycolaldehyde (GAH), in a reaction catalyzed by fructose-bisphosphate aldolase (*FBA1*), as reported by Bais et al. (1985); Chomvong et al. (2016); and Uranukul et al. (2019). Thus, we speculate that the additional GA observed might be produced from the phosphorylation of the XR-XDH pathway intermediate D-xylulose, thanks to the action of PFK and *FBA1*, similarly to what happens in *S. cerevisiae* (Fig. 4). Additionally, production of the intermediate GAH from the Weimberg/Dahms catabolism of D-xylose has been well described for bacterial aldolases (Boer et al. 2019; Ren et al. 2022).

Finally, no growth of *R. toruloides* was observed when EG was provided as the sole carbon source (Fig. 3c). Despite the very low amount of cells at the end of the fermentation (144 h), 0.224 ± 0.002 g L^−1^ of GA were detected (molar yield of 96.48% mol mol^−1^).

### Ethylene glycol catabolism in the presence of glucose and xylose

This study was designed to evaluate *R. toruloides* as a potential cell factory for the concomitant upcycling of lignocellulosic and PET hydrolysates. For this reason, EG metabolism was also assayed in the presence of both glucose and xylose. Similarly to the experiments with the single carbon sources, a C/N ratio of 80 was selected to induce lipid accumulation; additionally, a condition without nitrogen limitation (C/N of 8.8) was included. A buffer system for pH maintenance was used.

**Fig. 5:**
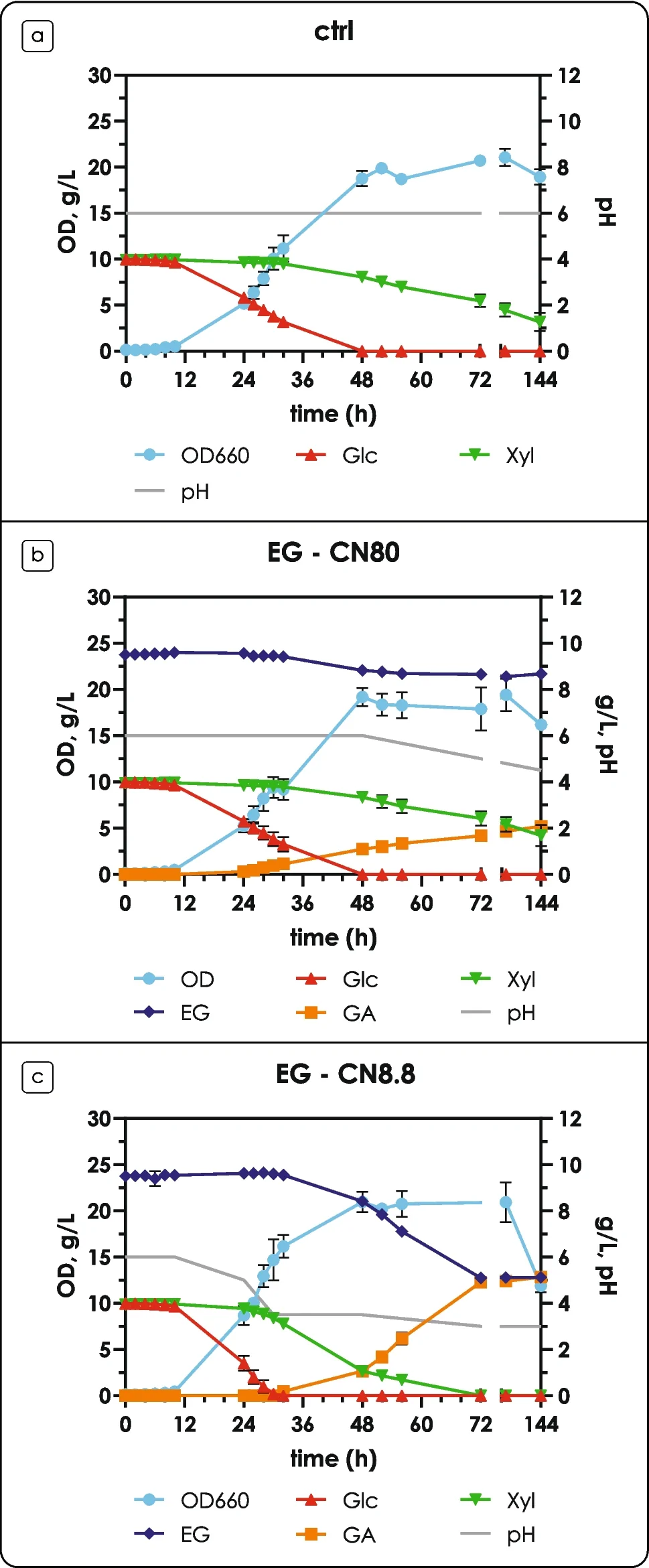
Fermentation profiles on glucose and xylose in the presence of ethylene glycol (EG) in shake flasks. (a) Fermentation profiles in the presence of glucose and xylose (C/N = 80) for the control condition. (b) Fermentation profiles in the presence of glucose and xylose (C/N = 80), and EG. (c) Fermentation profiles in the presence of glucose and xylose (C/N = 8.8), and EG. The left y-axis shows OD (blue, circles), glucose (red, upwards triangles), xylose (green, downwards triangles) and EG (dark blue, diamonds) concentration in g L^−1^; the right y-axis shows GA (orange, squares) concentration in g L^−1^ and pH (gray line). Glc: glucose; Xyl: xylose. Values are the mean ± standard deviation of three independent experiments

The fermentation profiles of cells grown in the presence of EG with a C/N ratio of 80 showed no significant differences with the control condition (no EG), apart from a drop in the pH starting from 48 h, due to the production of GA (Fig. 5a, b). As it was previously observed, EG consumption and GA production happened during glucose consumption, after the onset of the Nlim phase; production of GA continued after glucose was depleted and xylose started being consumed (Fig. 5a, b) in agreement with the previous results in bioreactor experiments. A different behavior, however, was observed with a C/N ratio of 8.8. EG consumption and GA production did not start until glucose was present in the medium, (Fig. 5c) in agreement with the previous results with glucose as a sole carbon source at C/N of 8.8 (Supplementary Fig. S2); after glucose depletion (30 h), GA production started. It is interesting to note that glucose inhibited EG consumption even if xylose was co-consumed, suggesting a catabolite repression mechanism different to the one observed in nitrogen-limiting conditions; Supplementary Fig. S6a shows a focus of the fermentation profiles around 24–48 h. Consistent with the above results, molar yield of GA from EG at nitrogen starvation was 117 ± 5% mol mol^−1^ and 191 ± 11% mol mol^−1^, on glucose and xylose, respectively (Supplementary Fig. 6b). Notably, the yield was below 100% (91 ± 4% mol mol^−1^) when the cells produced GA during xylose consumption without nitrogen limitation (Supplementary Fig. 6b).

### Putative pathway for EG metabolism in *R. toruloides*

In this work, we demonstrated that *R. toruloides* is able to produce GA from EG. To identify the pathway of EG metabolism, we performed an in silico analysis to seek which *R. toruloides* genes might be responsible. While yeast genes have yet to be identified and confirmed, there are reports in literature of a few bacterial genes which might be involved in the pathway. The most studied enzymes are from *Pseudomonas putida*; namely, encoded by *pedE* and *pedH* catalyzing the oxidation of EG to GAH, and *pedI* and *PP_0545* catalyzing the oxidation of GAH to GA (Franden et al. 2018). These enzymes require a complex cofactor (pyrroloquinoline quinone), which is not found in yeasts. For this reason, these genes were not included in our search, as orthologs in *R. toruloides* most likely do not exist.

A few other candidates have been proposed for the oxidation of EG to GAH. For bacterial genes, the most studied is *gox0313* from *Gluconobacter oxydans* (Zhang et al. 2015); *fucO* and *yqhD* from *Escherichia coli* have also been proposed; however, it has recently been shown that they most likely act as aldehyde reductases, catalyzing the opposite reaction (Yan et al. 2024). The alcohol oxidase *AOX1* from *Komagataella phaffii* (Isobe and Nishise 1994) and *YLL056 C* from *S. cerevisiae* (Senatore et al. 2024) have also been proposed as candidates. In addition, *GRE2* is also reported to be involved in EG metabolism, but in the reduction of GAH to EG (Jayakody and Jin 2021). In the study of Senatore and colleagues (Senatore et al. 2025), *YLL056C* was identified as a gene coding for an aldehyde reductase, similarly to *fucO*, *yqhD* and *GRE2*. For the oxidation of GAH to GA, *aldA* from *E. coli* (Yan et al. 2024), and *ALD2*, *ALD3*, *ALD4*, *ALD5* and *ARI1* from *S. cerevisiae* (Senatore et al. 2024) have been proposed.

Protein sequences of all the above-mentioned genes were retrieved from NCBI databases and blasted against *R. toruloides* IFO0880 protein database (Rhoto_IFO0880_4_GeneCatalog_proteins_20170509.aa), and *R. toruloides* NP11 and taxid:5286 protein databases using blastp. Considering only hits with a percent identity > 40%, a total of 970 hits were obtained, but the vast majority (96.4%) were unannotated. The obtained results are listed in **Supplementary Table** S4. Aox1, FucO and YqhD did not result in hits with a percent identity > 40%. The expression of the recovered hits was manually queried from available transcriptomics and/or proteomics data; it should be noted that omics data cited throughout this paragraph have been obtained in the control condition, i.e., in the absence of EG.

For the oxidation of EG to GAH, Gox0313 ortholog, alcohol dehydrogenase 15438 has been found highly abundant and upregulated at protein level on glucose during stationary or Nlim phase (Tiukova et al. 2019a; Kim et al. 2021; Reķēna et al. 2023). Also on transcriptome level, gox0313 ortholog was found highly abundant (2nd quartile) (Kim et al. 2021). The Yll056c orthologs, oxidoreductases belonging to NAD-dependent epimerase/dehydratase family, 9601 and 9602 or their orthologs, were detected at low levels or not present in available omics datasets. Similarly to Kim et al., we found the same hits for *S. cerevisiae GRE2* and *ARI1* activity. One of the two, hydroxysteroid dehydrogenase/isomerase 11160, was upregulated during the stationary phase on glucose on transcriptome and proteome level (Kim et al. 2021). The other NAD-dependent epimerase/dehydratase 12151 was not detected. It should be noted, however, that Gre2 in *S. cerevisiae* catalyzes the reduction of GAH to EG. Based on this survey, we speculated that for the oxidation of EG to GAH, alcohol dehydrogenases and hydroxysteroid dehydrogenase/isomerase might be involved.

In search for the genes responsible for the oxidation of GAH to GA in *R. toruloides*, only wide spectrum aldehyde dehydrogenases were detected by our BLAST analysis (**Supplementary Table** S4). From there, NAD-dependent aldehyde dehydrogenase 12042, an ortholog to *ScALD3*, has been found among top100 most abundant enzymes on glucose on the proteome level in *R. toruloides* IFO0880 (Kim et al. 2021), upregulated on stationary phase, and top250 on transcriptome level, only slightly increased during stationary phase. In *R. toruloides* CCT 7815, the ortholog of 12042 was highly abundant on the proteome level (top250) and upregulated during Nlim phase on glucose (Reķēna et al. 2023). In *R. toruloides* NP11, the ortholog of 12042 was highly abundant (1st quartile) on the proteome level on glucose during the exponential phase and only slightly higher during the Nlim phase (Tiukova et al. 2019a). Another NAD-dependent aldehyde dehydrogenase, an ortholog to 13426, was found highly abundant (1st quartile) and upregulated on the proteome level on glucose in *R. toruloides* CCT 7815 (Reķēna et al. 2023). Additionally, our results identified the NADPH-dependent methylglyoxal reductase (10497) as an ortholog to *ScARI1*, along with the above-mentioned 11160 and 12151. 10497 has been found upregulated on the proteome level on glucose in *R. toruloides* IFO0880 in the stationary phase (Kim et al. 2021) and in *R. toruloides* CCT 7815 during Nlim phase (Reķēna et al. 2023), respectively. Interestingly, according to the Supplementary of Kim et al., 10497 was an ortholog to *GRE2*, suggesting blurred lines among the potential candidates for oxidation of EG to GAH and GAH to GA. Another aldehyde dehydrogenase, ortholog to *E. coli* aldA, 11124, was upregulated in *R. toruloides* IFO0880 on glucose during stationary phase at transcriptome and proteome level (Kim et al. 2021). Its ortholog in CCT 8715 strain was upregulated on glucose during Nlim phase (Reķēna et al. 2023). A summary of this survey is presented in Supplementary Table S5. From this information, it seems that there are quite a few nonspecific aldehyde dehydrogenases that might be producing GA in *R. toruloides*.

The oxidation of GA to GOX is catalyzed by Gor1 (glyoxylate reductase) in *S. cerevisiae* (Rintala et al. 2007); while being reversible, the reaction is favored towards the formation of GA. The role of Gor1 in EG metabolism is still not clear: indeed, Senatore and colleagues (Senatore et al. 2025) showed that deletion of *GOR1* in *S. cerevisiae* did not cause any change in GA titer. In this study, we show that in *R. toruloides* GA is secreted in the environment rather than oxidized to GOX, nevertheless we looked for the presence of *GOR1.* First, a KEGG search with the keyword “glyoxylate” resulted in two activities, however, no genes were associated with IFO0880 (*Rhodosporidium toruloides* IFO0880 v4.0/FrozenGeneCatalog ver 1). To understand if IFO0880’s genome encodes for a glyoxylate reductase, Gor1 protein sequence from *S. cerevisiae* S288 C (*Sc*Gor1) was blasted against IFO0880’s protein database (Rhoto_IFO0880_4_GeneCatalog_proteins_20170509.aa) using blastp. The five hits obtained were aligned with *Sc*Gor1 using Clustal Omega (Supplementary Fig. S7, A and B). Protein Rhoto_IFO0880_4|12051 had the highest identity score (43.81%), and it was classified as the closest to *Sc*Gor1. Thus, we speculated that 12051 could be IFO0880’s glyoxylate reductase, as also could be sourced from the genome-scale model Rt_IFO0880 that we used to predict metabolic fluxes. To expand our search, 12051 was blasted (blastp) against two annotated protein databases from *R. toruloides* strains NP11 and taxid:5286. A total of 21 hits were obtained: hits with an identity score below 40% were discarded as non-homologs, resulting in five hits in total. The search identified a putative Gor1 in *R. toruloides* NP11, but the percent identity with 12051 was below 40%. These five hits, including 12051, *Sc*Gor1 and Gor1 from NP11 were aligned using Clustal Omega (Supplementary Fig. S7, C and D). 12051 showed identity above 90% with more than one hit (red dots in Supplementary Fig. S7, D); in particular, it showed 100% similarity with 2-hydroxyacid dehydrogenase and the putative protein GAA6048845.1_NBRC10513_003103 from taxid:5286. It also showed close to 100% similarity with 2-hydroxyacid dehydrogenase (DH) RHTO 05831 of the *R. toruloides* NP11; Repeatedly, 12051 showed only 31.45% identity with Gor1 from NP11.

**Fig. 6:**
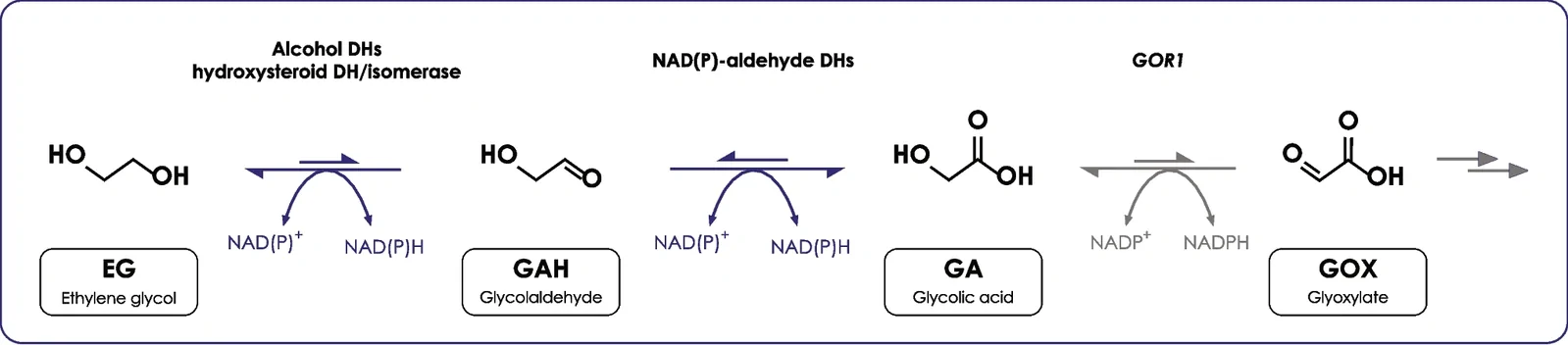
Proposed pathway for ethylene glycol (EG) metabolism in *Rhodotorula toruloides*. EG is first oxidized to glycolaldehyde (GAH) by alcohol dehydrogenases and/or hydroxysteroid dehydrogenase/isomerase, using NAD(P) as a cofactor. GAH is further oxidized to glycolic acid (GA) by the action of wide spectrum aldehyde dehydrogenases using NAD(P) as a cofactor. GA can be further oxidized to glyoxylate (GOX) by *GOR1*; the reaction is colored in gray as no flux was observed in this study. The length of the arrows for each reversible reaction suggests the favored product(s). More details can be found in the main text. Adapted from Senatore et al. (2024)

Gor1of *R. toruloides* NP11 has been detected as equally abundant with the 12051 by previous omics studies, notably without the presence of EG. Interestingly, 12051 was upregulated in *R. toruloides* IFO0880 during the stationary phase on glucose synthetic medium on the proteome level, but not on transcriptome level (Kim et al. 2021). In *R. toruloides* NP11, Gor1 was highly abundant (2nd quartile), but not upregulated during the Nlim phase on proteome level (Tiukova et al. 2019a). On the other hand, an ortholog to Gor1 in *R. toruloides* CCT 7815 (the study used NP11 annotation) was upregulated during the Nlim phase on glucose (2nd quartile) (Reķēna et al. 2023). NP11 2-hydroxyacid DH RHTO 05831 was also expressed in *R. toruloides* NP11 and CCT 7815 on the proteome level, but its abundance on glucose was low (4th quartile) (Tiukova et al. 2019a; Reķēna et al. 2023), making it less plausible candidate. This information is summarized in Supplementary Table S6. These results indicate that two genes that are not very similar to each other but can be found in different *R. toruloides* strains could serve as the Gor1 in *R. toruloides*. However, Gor1 activity could not be observed in our study, and a similar behavior was observed by Senatore and colleagues with *S. cerevisiae* (Senatore et al. 2025). Considering the overall results and observations, we propose the EG to GA conversion pathway reported in Fig. 6. The role of *GOR1* in EG metabolism remains unclear and further studies are required to elucidate the directionality of the reaction catalysed by Gor1 and Gor1 activity in the assayed conditions. Additionally, the proposed activities must be confirmed, considering that the behavior of alcohol (or aldehyde) dehydrogenases must be studied in vivo, as the redox state of the cell (and/or compartment) affects the directionality of the reaction; this will require the construction of multiple overexpression and deletion strains.

## Discussion

To the best of our knowledge, co-consumption of glucose and EG has only been reported once for yeasts (Kosiorowska et al. 2022): Kosiorowska and colleagues reported that *Y. lipolytica* is able to co-consume glucose and EG in rich medium (YPD); however, the addition of EG caused a strong reduction in growth and glucose uptake rate. GA is not mentioned as an EG conversion product and no explanation for the EG effect on *Y. lipolytica* growth is hypothesized. On the other hand, in *S. cerevisiae* cultures, EG consumption starts only after glucose, acetate and ethanol are depleted (Senatore et al. 2024). Quantitative physiology of *R. toruloides* EG metabolism in the presence of glucose revealed a very unusual phenotype: no significant differences could be observed compared to the control condition, for any of the measured parameters. Moreover, co-consumption of glucose and EG only happened during nitrogen starvation, suggesting that the activities involved in EG oxidation might be repressed during the exponential growth phase on glucose. This study also presents quantitative results on a significant drop in pH upon nitrogen starvation as a physiological characteristic for the *R. toruloides* species.

EG consumption in the presence of xylose resulted in molar yield of GA from EG of more than 100% during the Nlim phase, therefore we hypothesized alternative pathways for the synthesis of EG precursors. In *R. toruloides*, PFK is encoded by three genes (8859, 8863 or 8867/RHTO_00494), while FBA is encoded by a single gene (15420/RHTO_03043) (Zhu et al. 2012; Coradetti et al. 2018). As expected, PFKs and FBA are reported to be highly abundant in the presence of xylose by different strains (both during exponential and Nlim phases), and in some strains upregulated during lipid accumulation (Pinheiro et al. 2020; Kim et al. 2021). However, PKF specificity for D-xylulose was never described in *R. toruloides* (Tiukova et al. 2019b; Kim et al. 2021). In support of the putative pathway hypothesis (Fig. 4), no presence of glycolaldehyde (GAH) or GA was observed in the HPLC chromatograms in the control condition. These results suggest that EG is required for this phenotype, likely due to its contribution to the redox balance or, alternatively, by playing a role in gene expression regulation. From the results of genome-scale modeling, it could be inferred that the redox balance plays an important role in the EG metabolism in *R. toruloides*. The results demonstrated carbon channeling through the glyoxylate cycle towards GA biosynthesis. However, the activity of the NAD(P)-dependent *GOR1* remained unclear due to: (i) a futile cycle predicted on both control conditions with xylose or glucose (without the EG), and (ii) very high flux variability around those reactions in all simulations. Interestingly, the normalized flux through *GOR1* was higher on glucose compared to xylose, even though on glucose no carbon was metabolized into biomass, suggesting the role of EG in the redox balance and the fact that EG consumption leads to re-distribution of carbon irrespective of the GA yield on EG. The disagreement between predicted and measured exchange fluxes could point to the lack of more exchange fluxes to be measured (for example, gas exchange rates) or incomplete stoichiometries of the metabolic model. Possibly, these inconsistencies are related to the energy metabolism, as the futile cycles for reactions involving NAD(P)H were observed even when the pFBA method was applied. Further studies — transcriptomics, proteomics and metabolomics — for verification of these hypotheses are required.

Reported (molar) yields of GA from EG are always below 100% for yeast bioprocesses (Carniel et al. 2023; Senatore et al. 2024), suggesting that GA might be further metabolized to glyoxylate (GOX) (Fig. 5). In yeasts, GA can only be dissimilated to CO_2_ via the glyoxylate shunt, as they lack the key enzyme glyoxylate carboligase (Senatore et al. 2025), which catalyzes the condensation of two molecules of GOX to tartronate semialdehyde and CO_2_. The lack of growth of *R. toruloides* observed when EG was provided as the sole carbon source (Fig. 3c), the molar yield close to 100%, and the results obtained when cells were grown on glucose or xylose are all in line with this hypothesis: *R. toruloides* is not able to utilize EG as a carbon source, but only as an energy source.

One key aspect that makes *R. toruloides* stand out from the other yeasts is the yield of GA on consumed EG, which was 100% in the presence of glucose. This unusual result prompted us to identify gene(s) coding for glyoxylate reductase (*GOR1*) in *R. toruloides*. Our in silico analysis suggested a few candidate genes for glyoxylate reductase activity in different *R. toruloides* strains. It is worth noting that while according to existing proteomics databases these genes are expressed during lipid synthesis, it remains unclear why GA is not oxidized to GOX. This behavior seems to be specific to *R. toruloides*, as other yeasts show lower yields (Carniel et al. 2023; Senatore et al. 2024). Moreover, no other reaction consuming GA as a substrate has been reported in bacteria or yeast, suggesting that GA must be oxidized to GOX if the yield is not 100%. Further research is needed to elucidate the role of *GOR1* in EG metabolism.

To further explore the ability of *R. toruloides* to metabolize EG, consumption in the presence of xylose, or no additional carbon source was investigated as well. To the best of our knowledge, this is the first study reporting the ability of a microorganism to co-consume EG and xylose, during the exponential growth phase. Moreover, our results suggest that the presence of EG might represent an advantage, as EG catabolism might regenerate NAD(P)H and alleviate the characteristic cofactor imbalance of xylose metabolism. While further characterization in controlled cultivation conditions with *R. toruloides* is required to elucidate these hypotheses, based on the presented results EG supplementation could also be a strategy to improve xylose utilization in other yeasts, such as the well-known *Scheffersomyces stipitis*, without relying on (often complicated) metabolic engineering.

A very unique behavior was observed in terms of GA yield on consumed EG. In nitrogen-limiting conditions, co-oxidation of glucose and EG resulted in a yield of GA of about 100%. While the yield may seem unusually high, similar results have been observed with other yeasts: for instance, *S. cerevisiae* showed a yield of 94% mol mol^−1^ on consumed EG in a bioconversion approach (Senatore et al. 2024), and *S. stipitis* showed a yield of 95% mol mol^−1^ in a 10 L fermentation process for the production of GA from EG (manuscript in preparation). Co-oxidation of xylose and EG in nitrogen-limiting conditions consistently resulted in GA yields from consumed EG higher than 100%, with or without the presence of glucose as carbon source. This behavior was not observed in non-limiting nitrogen conditions, where the yield was around 90% mol mol^−1^, suggesting an involvement of glyoxylate reductase. Stable isotope labeling combined with mass spectrometry, as well as metabolic flux analysis are necessary to accurately characterize the phenotype observed in this preliminary study.

Finally, in silico analyses integrated with the available omics data allowed us to identify a set of genes putatively involved in EG metabolism in the species *R. toruloides*. To the best of our knowledge, this is the first report of such analysis for yeasts. While further confirmations on the proposed genes are required, the identification of fungal genes involved in EG metabolism remains crucial, as they often require NAD(P) as a cofactor and thus can be easily expressed heterologously, as opposed to the *P. putida ped* genes, requiring PQQ. Currently, the only NAD-dependent enzyme reported to be active on EG is the bacterial Gox0313.

Taken together, our results suggest that *R. toruloides* is an optimal candidate for the production of GA from EG. Additionally, its robustness and the improved fitness during growth on xylose in the presence of GA could be coupled to develop more efficient hemicellulosic-based biorefinery platforms. Indeed, *R. toruloides* could be exploited simultaneously for the sustainable production of microbial oils from residual biomasses and the valuable by-product GA from (waste) EG, while benefiting improved growth.

## Supplementary Information

Below is the link to the electronic supplementary material.

Supplementary file1 (PDF 12483 KB)

Supplementary file2 (XLSX 627 KB)

## Data Availability

The authors declare that the data supporting the findings of this study are available within the paper and its Supplementary Information files.
