# Supplementary material for: Ethylene glycol metabolism in the oleaginous yeast Rhodotorula toruloides
Source: Appl Microbiol Biotechnol. 2025 May 8;109:114. doi: 10.1007/s00253-025-13504-3 (PMC12062128; doi:10.1007/s00253-025-13504-3)
Supplement: Supplementary file 1 [file 253_2025_13504_MOESM1_ESM.pdf]

SUPPLEMENTARY INFORMATION

Ethylene glycol metabolism in the oleaginous yeast *Rhodotorula toruloides*

Vittorio Giorgio Senatore[1,†], Alīna Reķēna[2,†], Valeria Mapelli[1], Petri-Jaan Lahtvee[2], Paola Branduardi[1,*]

[1] Department of Biotechnology and Biosciences, University of Milano-Bicocca, Piazza della Scienza 2, 20126 Milan, Italy
[2] Tallinn University of Technology, Department of Chemistry and Biotechnology, Akadeemia tee 15, Tallinn, Estonia
[†] Vittorio Giorgio Senatore and Alīna Reķēna contributed equally to this work.

*Corresponding author: paola.branduardi@unimib.it

Keywords
Rhodotorula toruloides
Glycolic acid
Ethylene glycol
Glucose
Xylose
Polyethylene terephthalate

Graphical Abstract

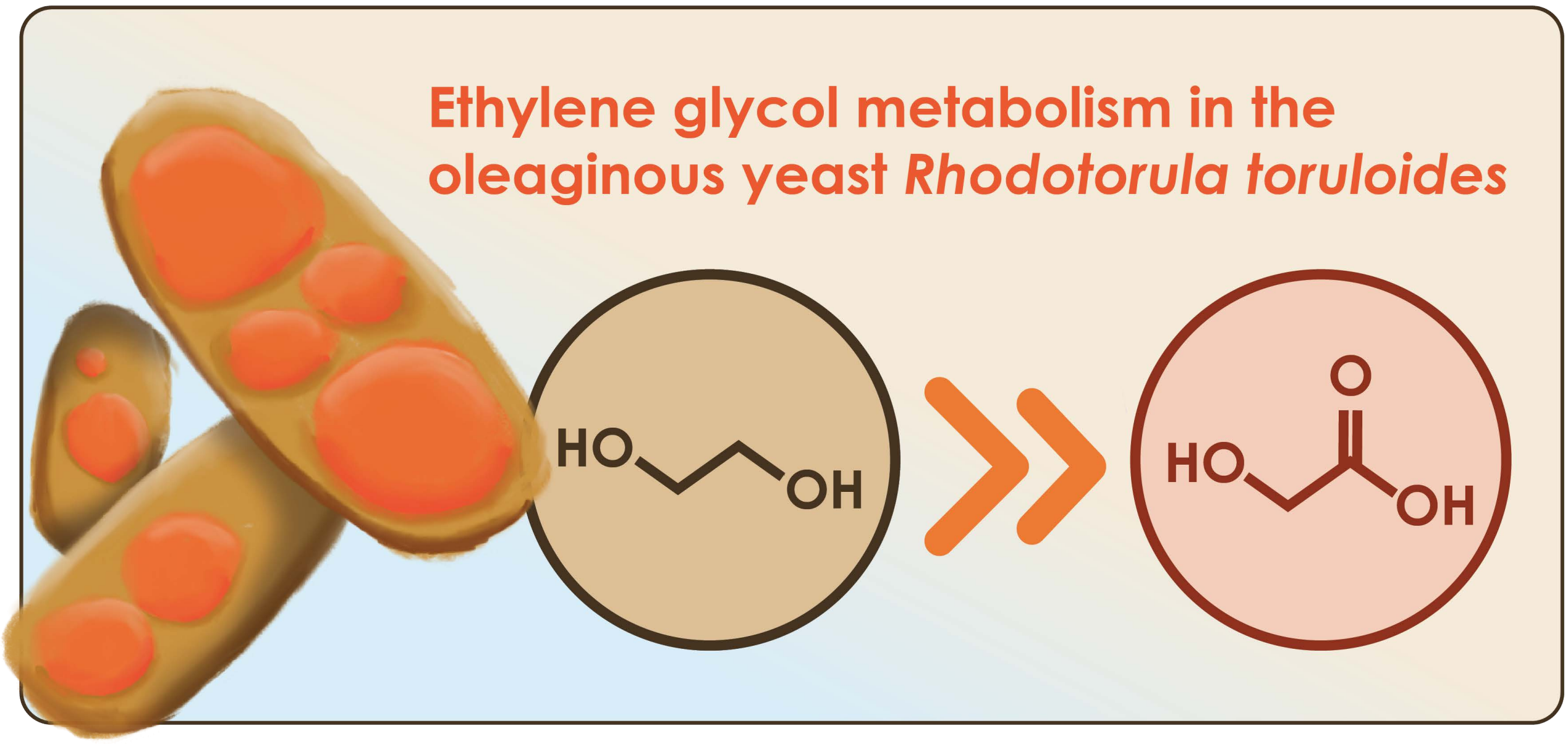

Table S1

Table S1 can be found in the supplementary file "Supplementary Tables.xls".

Table S2

Table S2 can be found in the supplementary file “Supplementary Tables.xls”.

Figure S1

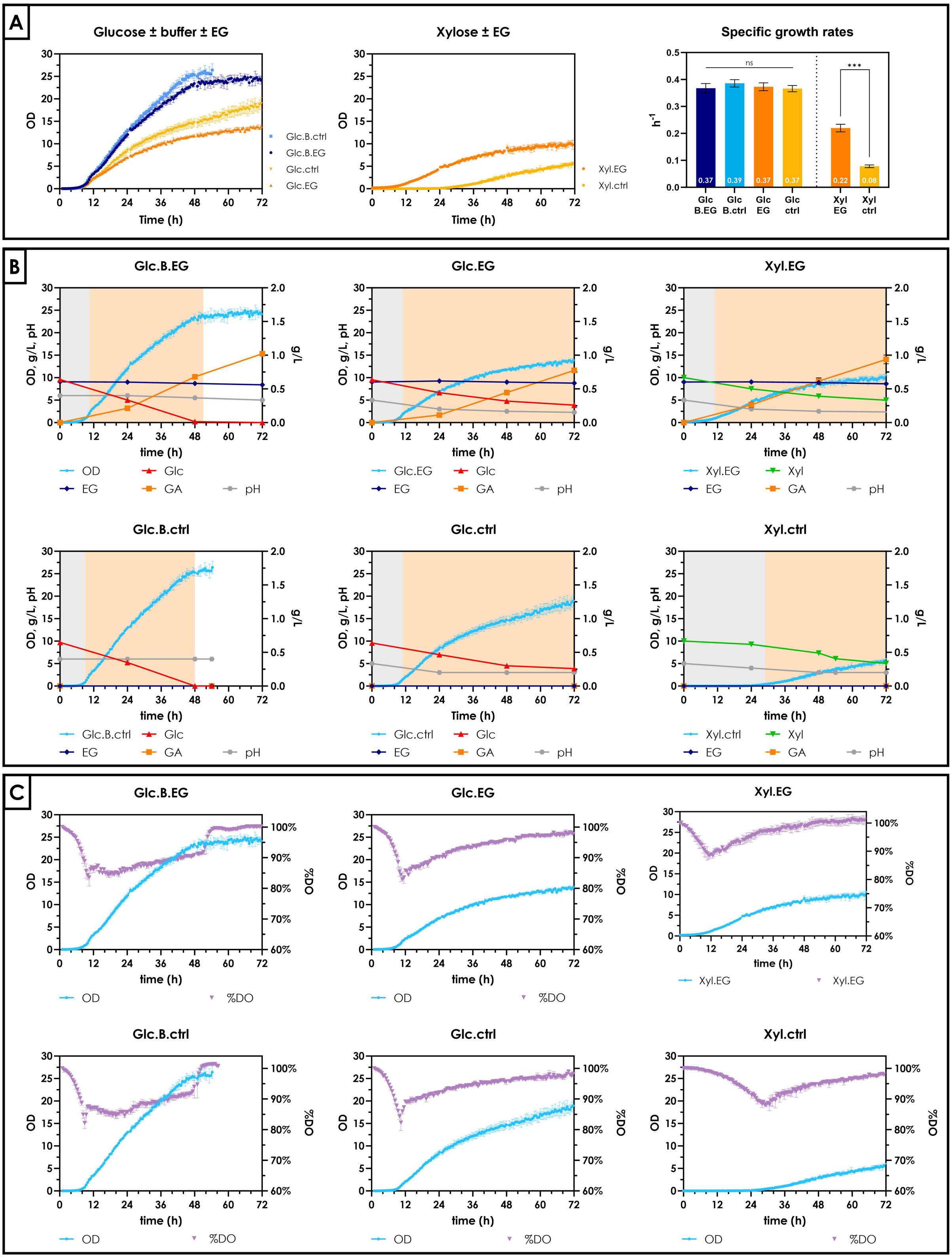

Fig. S1 Fermentation profiles on glucose or xylose in the presence of ethylene glycol (EG) in RTS-8 plus multichannel bioreactor. Panel A shows the OD live data and the maximum specific growth rates in the conditions assayed. Panel B shows the full fermentation profiles; the left y-axis shows pH (gray line), OD (blue), glucose (red, upwards triangles), xylose (green, downwards triangles) and EG (dark blue, diamonds) concentration in g L^{-1}; the right y-axis shows GA (yellow, diamonds) concentration in g L^{-1}. The exponential phase is shaded in gray, the Nlim phase is shaded with orange, the phase after depletion of glucose is unshaded. Panel C shows the percentage of dissolved oxygen (%DO) and OD values for the assayed conditions. Values are the mean ± standard deviation of three independent experiments.

Figure S2

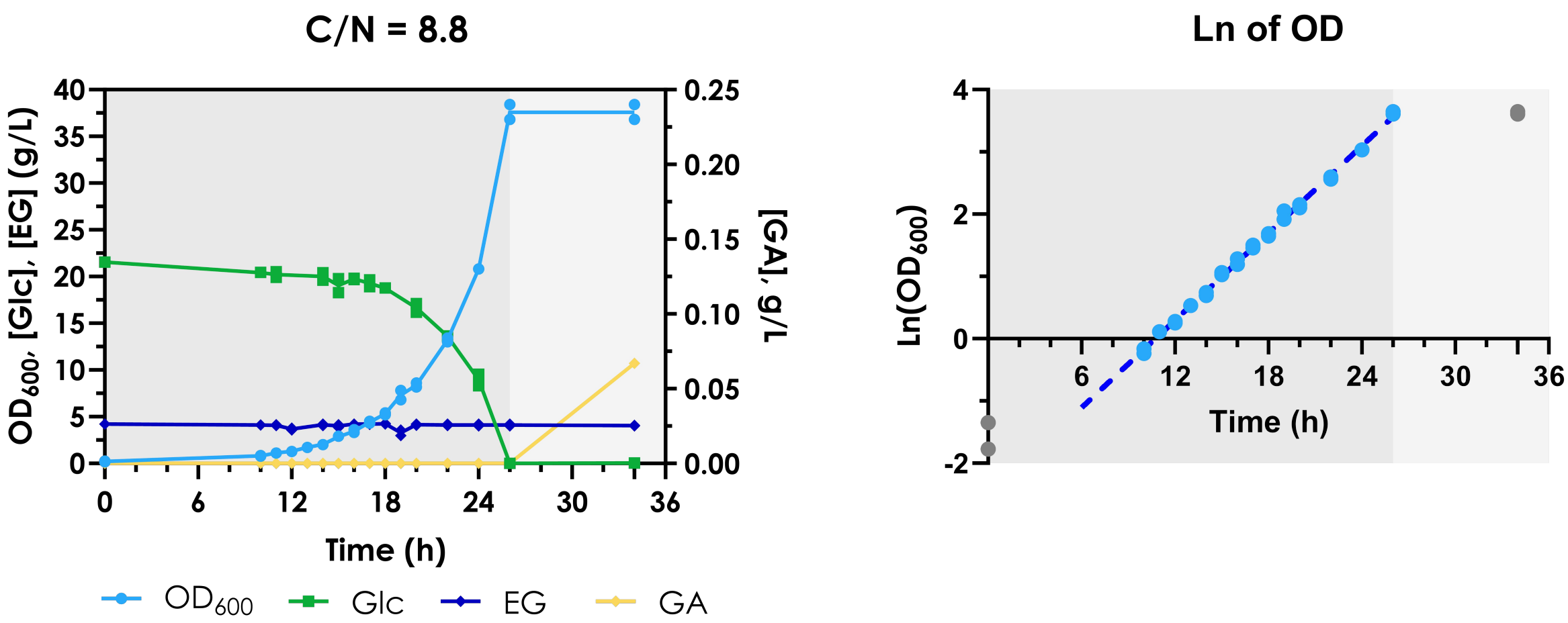

Fig S2. Fermentation profile of *R. toruloides* grown on 20 g L^{-1} glucose, C/N of 8.8 in the presence of ethylene glycol (EG). The left panel shows the fermentation profile obtained without nitrogen limitation. As in shake flasks experiments, EG consumption and GA production do not start until the end of the exponential phase, which corresponds to the depletion of glucose, in this case. The left y-axis shows OD (blue, circles), and glucose (green, squares) and EG (dark blue, diamonds) concentration in g L^{-1}; the right y-axis shows GA (yellow, diamonds) concentration in g L^{-1}. The right panel shows the growth rate during the exponential growth phase. The y-axis shows the natural logarithm of the OD and the calculated regression line; the slope of the line was used to represent the growth rate; points that were excluded from the fitting are shaded in grey. Values of each independent experiment (2) are plotted; the line connects the mean of each value.

Figure S3

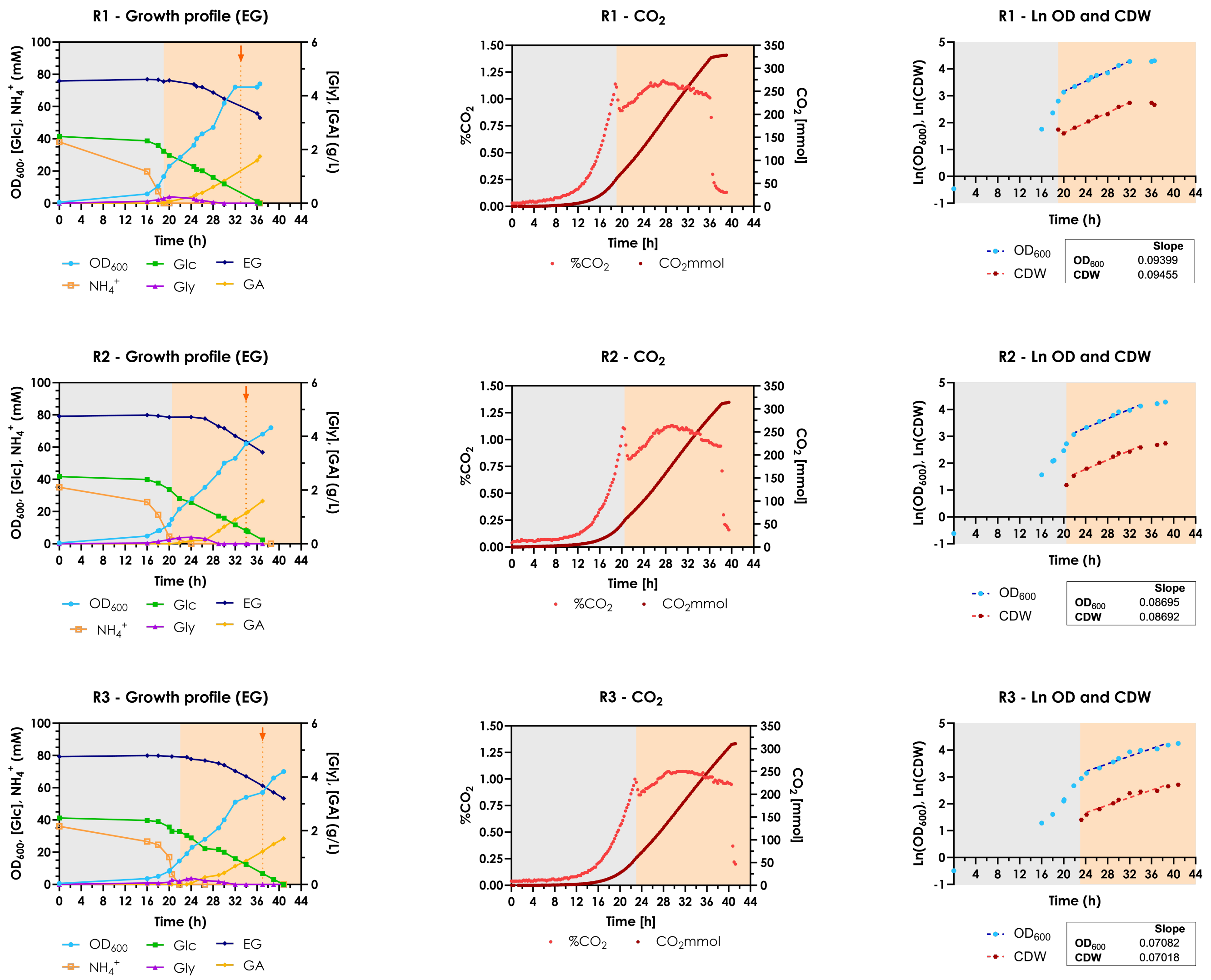

Fig S3. Fermentation profiles in the presence of glucose and ethylene glycol (EG). The left column shows the fermentation profiles; the left y-axis shows OD (blue, circles), glucose (green, squares) and EG (dark blue, diamonds) concentration in g L^{-1}, and ammonium concentration in mM; the right y-axis shows glycerol (purple, diamonds) and GA (yellow, diamonds) concentration in g L^{-1}. The exponential phase is shaded in gray, while the Nlim phase is shaded with orange; the arrow indicates the time of sampling for total protein and lipid profile analyses. The central column shows the %CO_2 (light red) and accumulated CO_2 (dark red) in mmol. The right column shows growth rates with OD and CDW measurements; the slope of the regression lines was used to represent the growth rate.

Figure S4

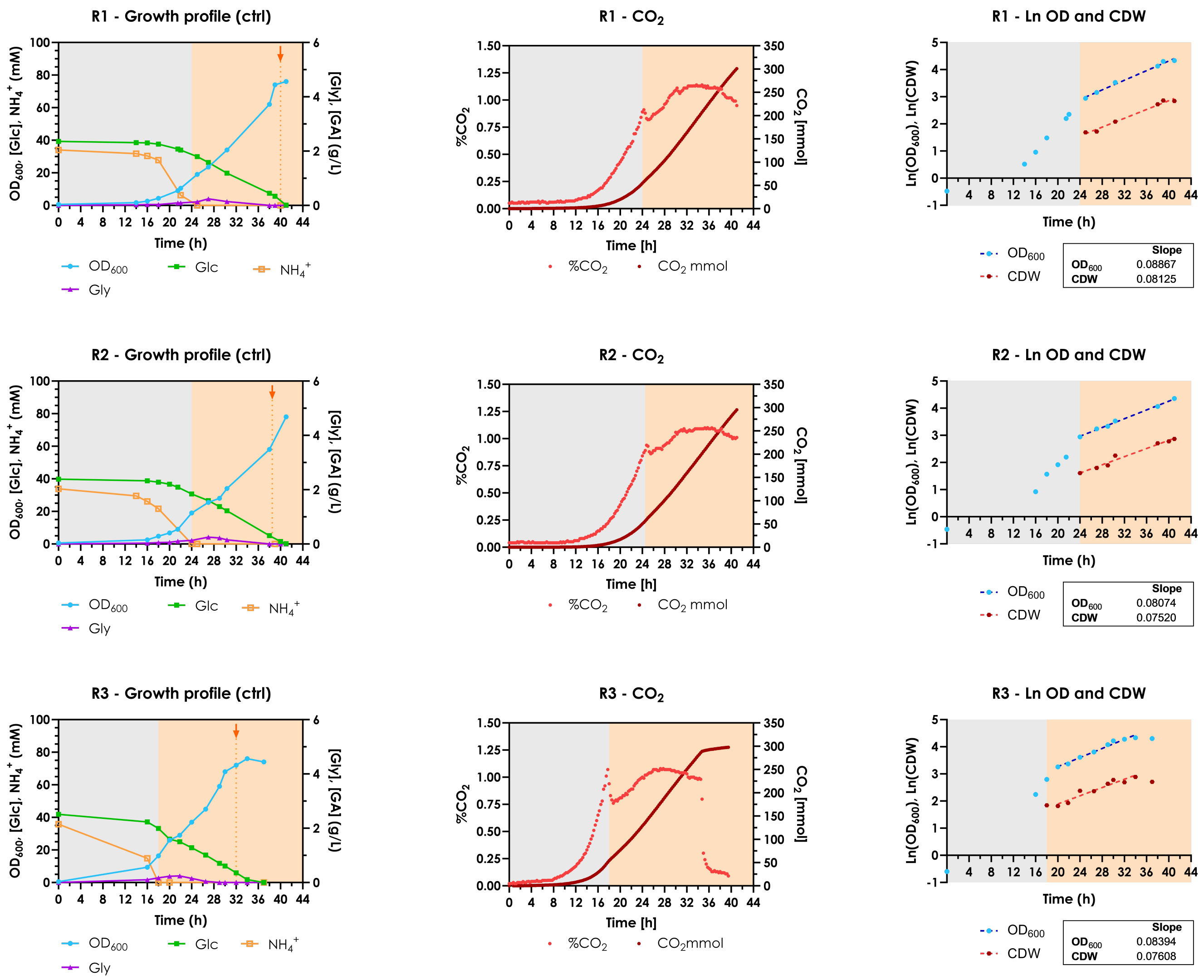

Fig S4. Fermentation profiles in the control condition. The left column shows the fermentation profiles; the left y-axis shows OD (blue, circles), glucose (green, squares) concentration in g L^{-1}, and ammonium concentration in mM; the right y-axis shows glycerol (purple, diamonds) and GA (yellow, diamonds) concentration in g L^{-1}. The exponential phase is shaded in gray, while the Nlim phase is shaded with orange; the arrow indicates the time of sampling for total protein and lipid profile analyses. The central column shows the %CO_2 (light red) and accumulated CO_2 (dark red) in mmol. The right column shows growth rates with OD and CDW measurements; the slope of the regression lines was used to represent the growth rate.

Figure S5

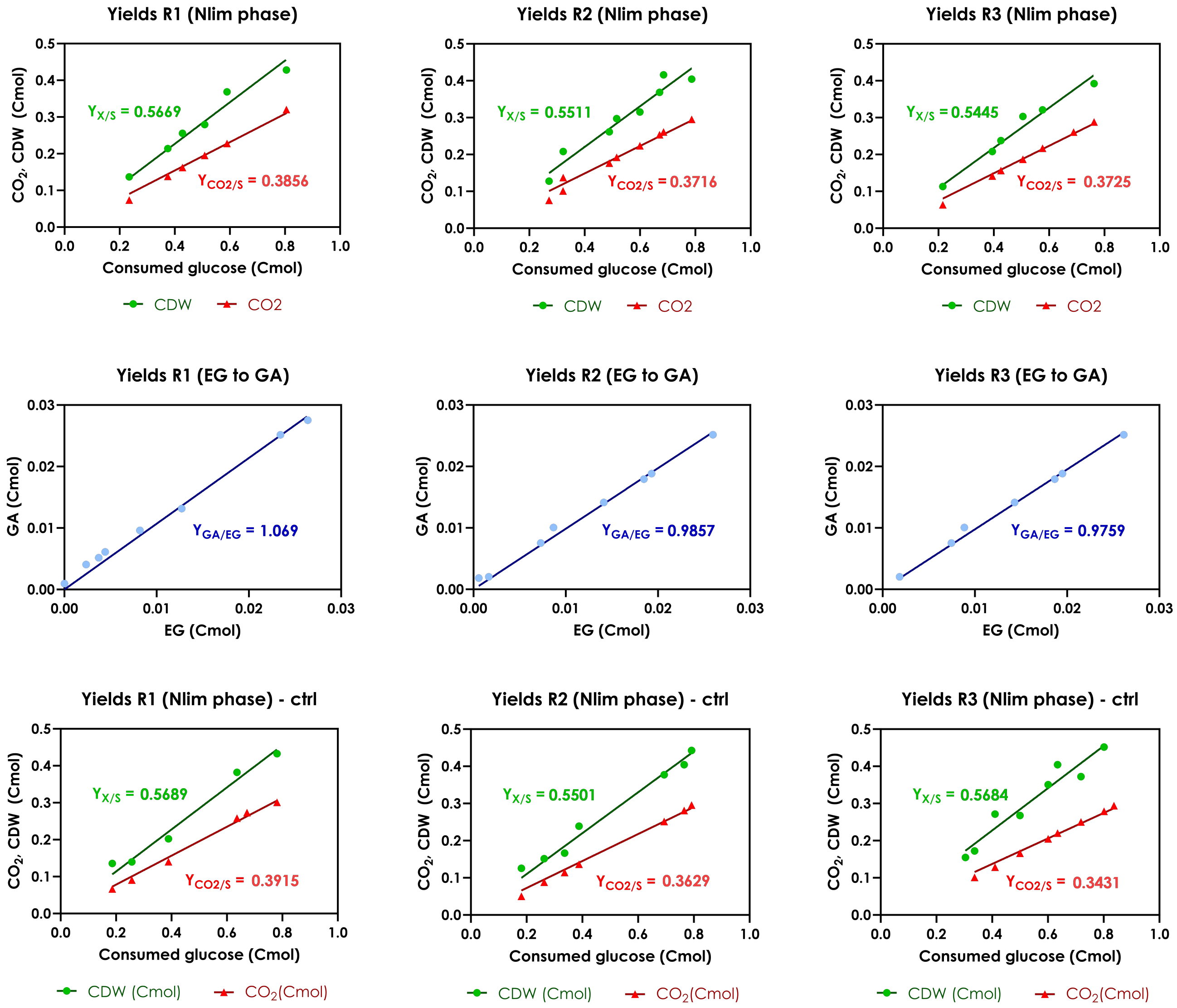

Fig S5. Carbon balance of the Nlim phase in the absence and presence of EG. The first row shows the yields in Cmol Cmol^{-1} (X/S and CO_2/S) in the presence of EG for each replicate. The second row shows the yield of glycolic acid on consumed ethylene glycol (Cmol Cmol^{-1}); the slope of the line was used to represent the yield. The last row shows the yields in Cmol Cmol^{-1} (X/S and CO_2/S) for the control condition.

Table S3

	Ctrl	EG
**Total lipid content**	31.49 ± 2.64	29.28 ± 2.58
**C11:0**	0.13 ± 0.01	0.13 ± 0.02
**C14:0**	0.89 ± 0.02	0.95 ± 0.01
**C15:0**	0.12 ± 0.01	0.08 ± 0.01
**C16:0**	24.15 ± 0.35	25.19 ± 0.32
**C16:1c9**	0.42 ± 0.02	0.48 ± 0.01
**C17:1c9**	0.18 ± 0.04	0.20 ± 0.01
**C18:0**	8.72 ± 0.37	8.41 ± 0.50
**C18:1t9**	0.09 ± 0.01	0.07 ± 0.01
**C18:1c9**	41.70 ± 0.66	40.93 ± 0.24
**C18:1c11**	0.14 ± 0.01	0.2 ± 0.01
**C18:2t9t12**	0.15 ± 0.04	0.18 ± 0.03
**C18:2n6**	16.42 ± 0.41	16.48 ± 0.46
**C20:0**	0.30 ± 0.01	0.29 ± 0.01
**C18:3n3**	4.57 ± 0.50	4.65 ± 0.69
**C20:1c11**	0.24 ± 0.02	0.22 ± 0.02
**C20:2n6**	0.12 ± 0.01	0.11 ± 0.01
**C22:0**	0.51 ± 0.03	0.47 ± 0.03
**C24:0**	1.14 ± 0.08	0.99 ± 0.04

Table S3. Lipid profile in the presence and absence of EG at the end of the Nlim phase. Total lipid content is expressed in g/100 g_{CDW}; abundance of each fatty acid is expressed as the percentage of the form on the total amount of lipids. Values are the mean ± standard deviation of three independent experiments. No significant differences (P-value > .05) were observed.

Figure S6

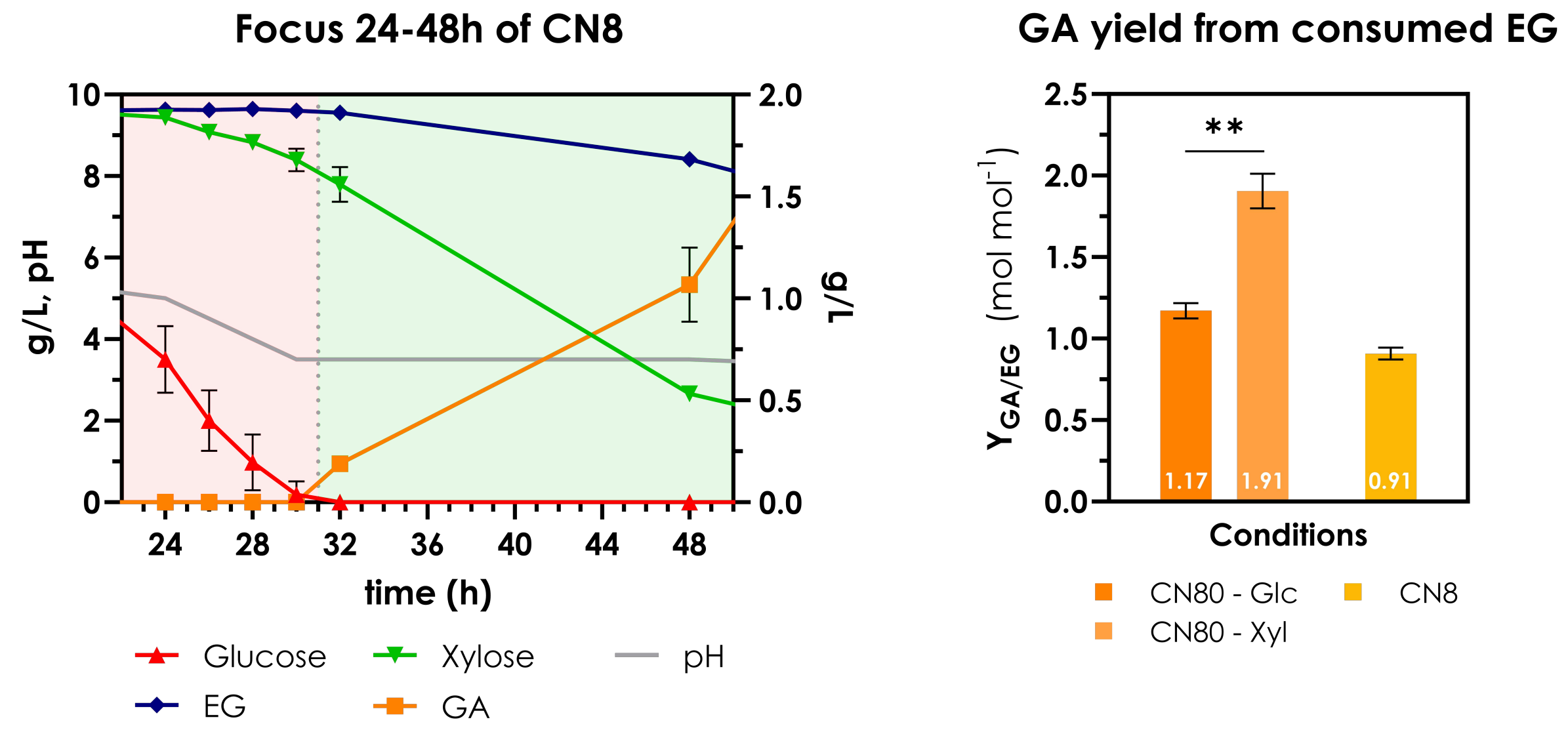

Fig. S6 Fermentations on glucose and xylose in the presence of ethylene glycol (EG) in shake flasks. The left panel shows a focus between 24 – 48 h of the fermentation profile of cells growing in the presence of both glucose and xylose (CN = 8), and EG. As it can be observed, EG consumption and GA production only start between 30 – 32 h, upon depletion of glucose. The left y-axis shows OD (blue, circles), glucose (red, upwards triangles), xylose (green, downwards triangles) and EG (dark blue, diamonds) concentration in g L^{-1}; the right y-axis shows GA (orange, squares) concentration in g L^{-1} and pH (gray line). Glc: glucose; Xyl: xylose. Values are the mean ± standard deviation of three independent experiments.

The right panel shows the calculated GA yields on consumed EG (mol mol^{-1}) in the presence of different growth phases and C/N ratios. The orange bars refer to the yields obtained with a C/N ratio of 80: the dark orange column (left) is the yield obtained in the glucose consumption phase; the light orange column (center) is the yield obtained in the xylose consumption phase. The dark yellow bar refers to the yield obtained with a C/N ratio of 8.8; in this case, GA production only happened during the xylose consumption phase. Values are the mean ± standard deviation of three independent experiments.

Figure S7

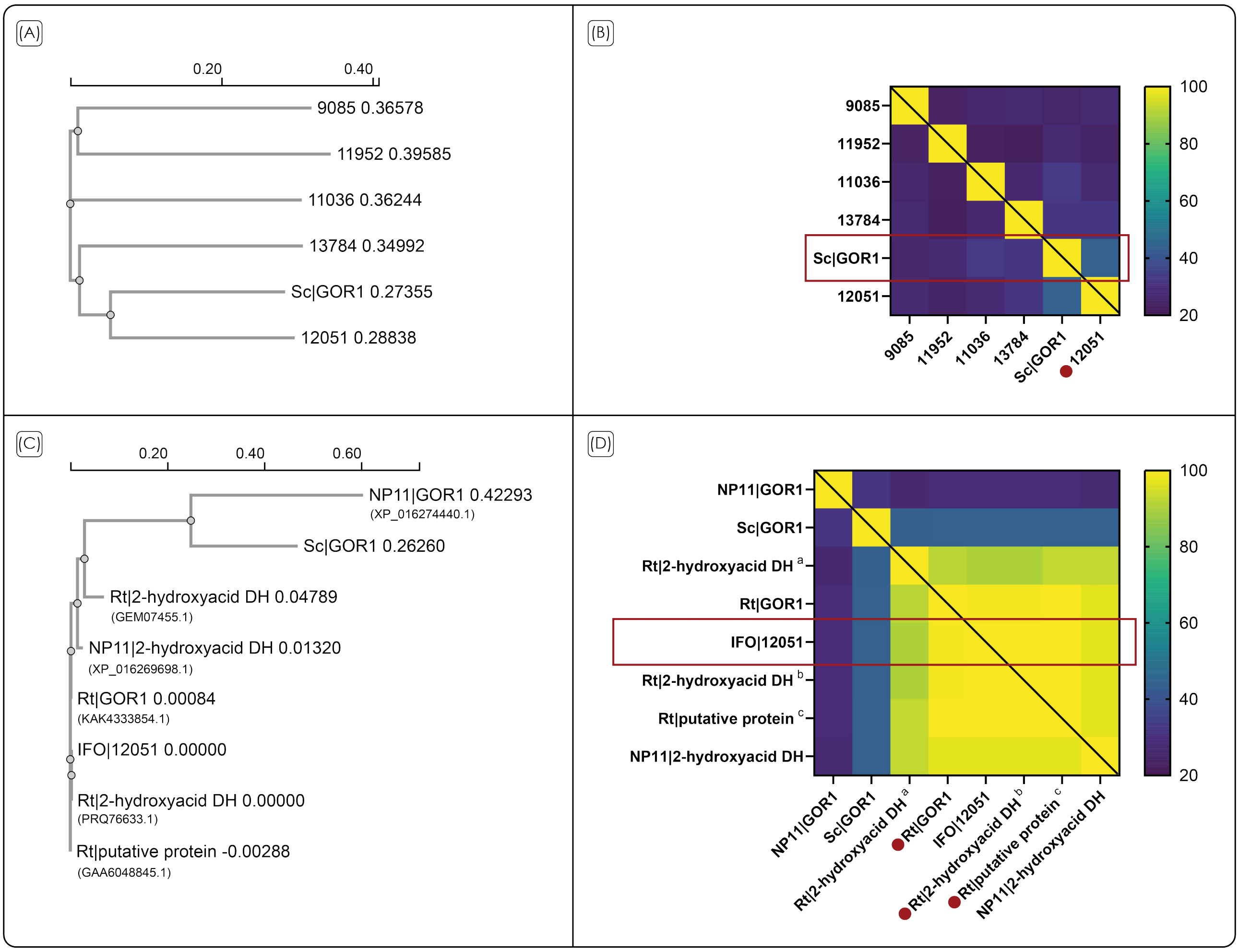

Fig S7. BLAST and alignment results of *S. cerevisiae* Gor1 with *R. toruloides* strains IFO0880, NP11 and taxid:5286. Panels A (phylogeny tree) and B (identity matrix): Gor1 protein sequence of *S. cerevisiae* S288C (*Sc*Gor1) was blasted against IFO0880's protein database Rhoto_IFO0880_4_GeneCatalog_proteins_20170509.aa using blastp. The five hits obtained were aligned with *Sc*Gor1 using Clustal Omega. Panels C (phylogeny tree) and D (identity matrix): 12051 protein sequence from IFO0880 was blasted using blastp against two annotated protein databases from *R. toruloides* strains NP11 and taxid:5286. Only the hits with a percent identity > 40% are shown; Gor1 from strain NP11 was included for completeness. Rt: *R. toruloides* taxid:5286; IFO: *R. toruloides* IFO0880. [a]: GEM07455.1; [b]: PRQ76633.1; [c]: GAA6048845.1_NBRC10513_003103.

Table S4

Table S4 can be found in the supplementary file “Supplementary Tables.xls”.

Table S5

Description	IFO0880	CCT 7815	NP11	IFO0880 stationary phase (a)		CTT 7815 Nlim phase (b)	NP11 Nlim phase (c)
				transcriptome	proteome	proteome	proteome
Go *gox0313* ortholog	15438	3062	3062	=	↑	↑	↑
Sc *Yll056c* ortholog	9601 and 9602	4070 and 4071	4070 and 4071	na	na	na	na
Sc *GRE2* ortholog	11160, 12151	4342 and 2276	4342 and 2276	↑, na	↑, na	=, na	=, na
Sc *ALD3* ortholog	12041, 13426	5838, 4425	5838, 4425	=,=	↑,=	↑,↑	=,=
Sc *ARI1* ortholog	11160, 12151, 10497	4342, 2276, 3406	4342, 2276, 3406	↑, na,=	↑, na,↑	=, na,↑	=, na,=
Ec *aldA* ortholog	11124	4310	4310	↑	↑	↑	=

Table S5. Transcriptomics and proteomics data available for gox0313, *YLL056C, GRE2, ALD3, ARI1*, and aldA. Upregulated genes are indicated with one upwards arrow (↑); highly upregulated genes are indicated with two arrows (↑↑); genes that are expressed, but not upregulated are indicated with an equal sign (=); genes that have not been identified are indicated with na (not applicable).
(a) - Kim et al., 2021; (b) - Reķēna et al., 2023; (c) - Tiukova et al., 2019.

Table S6

				IFO0880 stationary phase (a)		CTT 7815 Nlim phase (b)	NP11 Nlim phase (c)
**Description**	**IFO0880**	**CCT 7815**	**NP11**	transcriptome	proteome	proteome	proteome
Sc *GOR1* ortholog	12051			=	↑		
12051 ortholog		*GOR1*	*GOR1*			↑↑	=
12051 ortholog		RHTO 05831	RHTO 05831			=	=

Table S6. Transcriptomics and proteomics data available for *GOR1*. Upregulated genes are indicated with one upwards arrow (↑); highly upregulated genes are indicated with two arrows (↑↑); genes that are expressed, but not upregulated are indicated with an equal sign (=).
(a) - Kim et al., 2021; (b) - Reķēna et al., 2023; (c) - Tiukova et al., 2019
